# DNA Barcoding‐Enabled Tracking of Lipid Nanoparticles: Drug‐Loading‐Dependent Biodistribution and Tumor Microenvironment Targeting

**DOI:** 10.1002/adhm.202501914

**Published:** 2025-06-26

**Authors:** Letao Xu, Rui Chen, Xing Wang, Dawei Liu, Yun liu, Chun‐Xia Zhao

**Affiliations:** ^1^ School of Chemical Engineering Faculty of Science Engineering and Technology The University of Adelaide Adelaide SA 5005 Australia; ^2^ Australian Institute for Bioengineering and Nanotechnology The University of Queensland St Lucia QLD 4072 Australia

**Keywords:** DNA barcoding, drug delivery, drug loading, lipid nanoparticles, tumor microenvironment

## Abstract

Lipid nanoparticles (LNPs) are versatile drug delivery systems, yet the impact of drug loading (DL) on their biodistribution and cellular uptake remains poorly understood. Optimizing drug loading is crucial for enhancing therapeutic efficacy and safety, as higher loading allows for lower LNP doses, reducing overall nanomaterial burden. Addressing this knowledge gap is essential for advancing LNP‐based cancer therapies. This study integrates DNA barcoding technology with LNPs to evaluate their in vivo delivery behaviors under varying drug loadings. Using a sequential nanoprecipitation method, DNA‐barcoded LNPs with low (1%), medium (16%), and high (26%) drug loadings are fabricated, each tagged with a unique DNA barcode for precise tracking. Pooled LNPs are intravenously administered to tumor‐bearing mice, and their biodistribution across organs is quantified via qPCR. High drug‐loading LNPs demonstrate preferential accumulation in the spleen, while low drug‐loading LNPs exhibit higher liver accumulation, suggesting faster clearance. Cellular uptake analysis reveals enhanced uptake of high drug‐loading LNPs by tumor‐associated macrophages within the tumor microenvironment (TME). This study establishes a robust platform for simultaneous and high‐sensitivity monitoring of LNP behaviors, significantly reducing animal use and interanimal variability. The findings guide the rational design for developing optimal LNPs for cancer therapies targeting specific TME components.

## Graphical abstract

In this study, lipid nanoparticles are tracked in vivo using DNA barcodes to determine how varying drug loadings affect their delivery behaviors in mice. A cost‐effective strategy is developed for simultaneously monitoring the biodistribution of a library of lipid nanoparticles (LNPs). Importantly, LNPs with high drug loading show promise for future cancer immunotherapy, underscoring the importance of investigating drug‐loading effects.

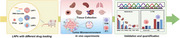

## 1 Introduction

Nanoparticles have garnered significant interest as drug carriers since the FDA approval of Doxil, a liposomal doxorubicin.^[^ ^1^ ^]^ Among the various types of nanoparticle‐based drug carriers, lipid nanoparticles (LNPs) are the most widely used, achieving remarkable success in both preclinical and clinical settings,^[^ ^2^ ^]^ owing to their intrinsic ability to encapsulate both hydrophobic and hydrophilic therapeutics.^[^ ^3^ ^]^ Furthermore, as drug delivery vehicles, LNPs offer numerous advantages, including great biocompatibility, ease of preparation, scalability, low immunogenicity, and potential for targeted delivery.^[^ ^4^ ^]^ Their continued success across diverse fields highlights their established role as next‐generation drug delivery systems.

Despite the success of LNP drug delivery systems, like most other organic nanocarriers, they generally exhibit relatively low drug loading (<10 wt%).^[^ ^5^ ^]^ As a result, achieving therapeutic levels in circulation often requires high drug dosages and large quantities of materials, which can increase the body's material burden and potentially induce toxicity or chronic inflammation.^[^ ^6^, ^7^, ^8^ ^]^ Given that drug loading (DL) is a crucial parameter for effective drug delivery, developing a straightforward and efficient method to boost the drug loading of nanocarriers remains a significant challenge.

We recently developed a concentration‐controlled sequential nanoprecipitation method capable of producing LNPs with tunable drug loadings, ranging from low to high levels (>70%). By adjusting the concentrations of lipids and drugs in the system, we modulated the precipitation sequence, ensuring that the drug precipitated first to form a drug core subsequently encapsulated in lipid particles. This method offers several key advantages, including procedural simplicity, high encapsulation efficiency, high stability, and well‐controlled in vitro release profiles.^[^ ^9^ ^]^ Although previous studies on polymeric and silica nanoparticles have demonstrated favorable therapeutic efficacy with higher drug loading, the in vivo nano–bio interactions that link drug loading to delivery behavior and therapeutic outcomes in LNPs remain unexplored.^[^ ^10^, ^11^ ^]^

While interest in high‐drug‐loading nanoparticles is growing, few studies have systematically compared the biodistribution and cellular tropism of nanoparticles with varying drug loadings. Such comparisons are essential for understanding how drug loading impacts nanoparticle performance. Even when nanoparticles share the same material compositions and basic physicochemical properties (e.g., size, charge), drug loading can alter other key characteristics that are not yet well understood, largely due to limitations of current knowledge and technology.^[^ ^12^ ^]^

Traditional comparative studies typically control a single variable, such as maintaining equal drug concentrations or nanoparticle counts, often leading to potentially “unfair” comparisons. Moreover, these studies frequently rely on in vitro studies to reduce labor, time, and animal costs. However, growing evidence highlights the weak correlation between in vitro and in vivo results, emphasizing the importance of assessing nanoparticle behavior in animal models rather than relying solely on in vitro studies.^[^ ^13^, ^14^ ^]^ This creates a pressing need for methods capable of simultaneously evaluating the in vivo delivery performance of nanoparticles with different drug loadings. To address this challenge, DNA barcoding technology has been integrated for multiplexed in vivo tracking of variants in a single experiment.^[^ ^15^, ^16^ ^]^ Each unique DNA barcode represents a specific formulation, allowing precise quantification of each formulation's performance through advanced technologies such as deep sequencing, qPCR, and digital droplet PCR (ddPCR). In combination with these advanced technologies, the DNA barcoding approach offers superior sensitivity and accuracy in tracking formulations compared to the conventional dye labeling approach, avoiding common issues such as quenching, leakage, and self‐aggregation.^[^ ^17^ ^]^ The integration of DNA barcoding has not only improved the reliability of nanoparticle tracking but also enables multiplexing, providing a robust approach for evaluating and achieving high‐throughput screening of formulations.

In this study, we incorporated unique DNA barcodes into three LNPs with different drug loadings and mixed them as a single pool. The pooled barcoding‐LNPs (b‐LNPs) were then administered to 4T1 tumor‐bearing mice, a triple‐negative breast cancer (TNBC) model, to monitor the biodistribution and tumor tropism of LNPs with different drug loadings. Furthermore, we employed qPCR and a TRIzol‐based DNA extraction method to quantify the delivery and accumulation of LNPs with different drug loadings in vivo simultaneously. This study introduces a cost‐effective strategy for investigating the delivery behaviors of LNPs with different drug loadings in vivo, establishing a cost‐effective and reliable method for routine laboratory analysis of nanoparticle behaviors, and is broadly applicable to various drug delivery systems beyond LNPs.

## 2 Results and Discussion

### 2.1 Screening of DNA Barcode‐Loaded LNPs

A concentration‐controlled sequential nanoprecipitation method was developed to produce LNPs with tunable drug loadings, ranging from low to exceptionally high, while maintaining well‐controlled physicochemical properties.^[^ ^9^ ^]^ To further explore the in vivo delivery behaviors of these LNPs with different drug loadings in tumor models simultaneously, we first incorporated distinct DNA barcodes into these formulations as probes. For initial screening, we prepared six b‐LNPs formulations with a fixed theoretical drug loading of 30% docetaxel (DTX), composed of ALC‐0315, DMPC, cholesterol, and DSPE‐PEG 2 KDa, respectively, and varied N/P ratios ranging from 1 to 6. In these formulations, ALC‐0315 was an ionizable lipid for DNA encapsulation, it has been FDA‐approved and used in the COVID‐19 mRNA vaccine BNT162b2.^[^ ^18^ ^]^ The N/P ratio represents the ratio of nitrogen (N) in positively charged amine groups to phosphorus (P) in negatively charged nucleic acid phosphate groups. Considering the potential immunogenicity risks associated with ionizable lipids, this screening procedure prioritized the synthesis of b‐LNP formulations with relatively high loading of hydrophobic drugs and low N/P ratios for encapsulation of DNA barcodes while maintaining a nanoscale size (≈100 nm) and high monodispersity. These factors collectively minimize the primary safety risks associated with the intravenous injection of nanomedicine.^[^ ^7^ ^]^ Additionally, stability in size and encapsulation efficiency for the b‐LNPs were also considered important factors during the screening process.

**Figure 1:**
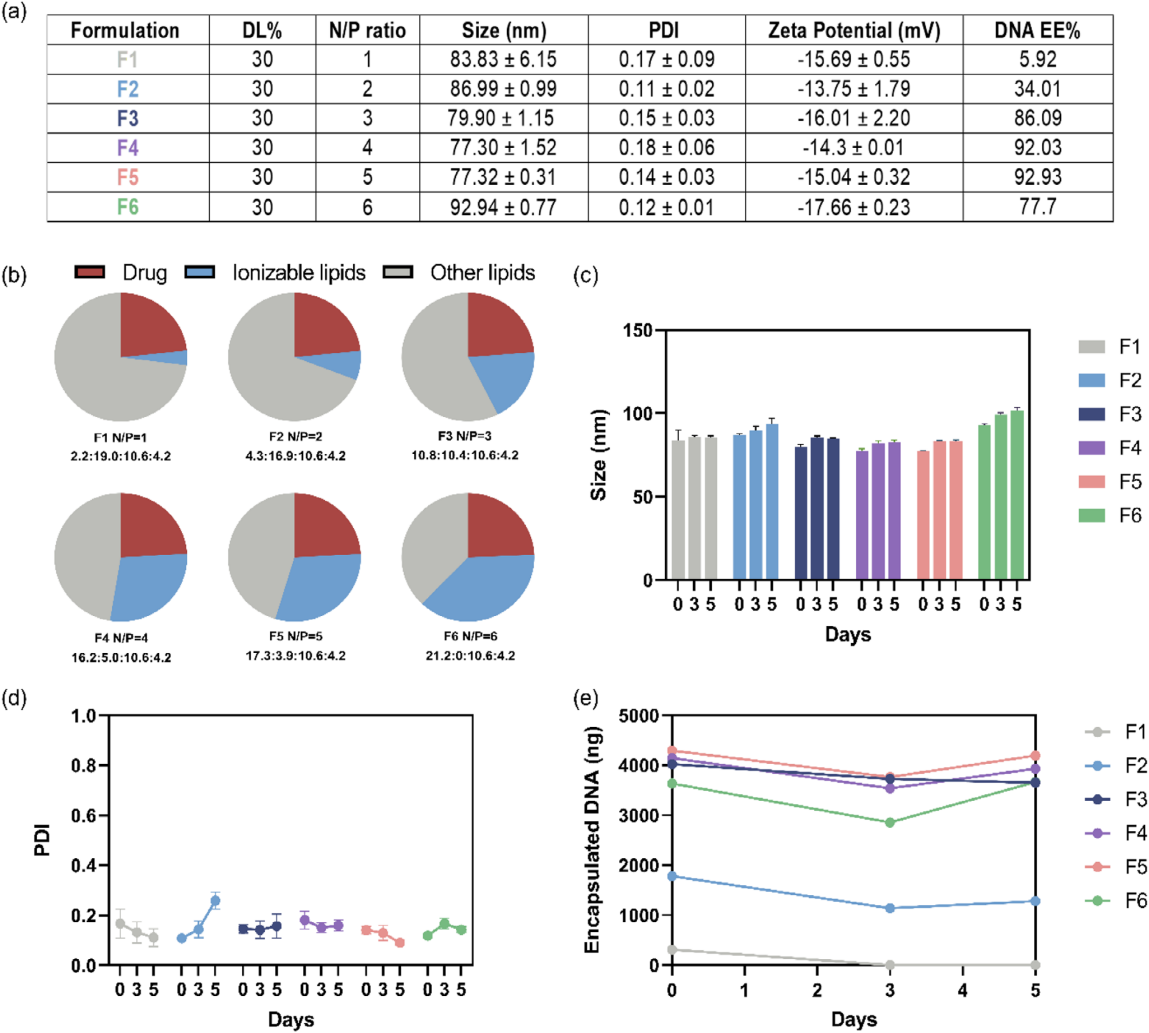
Screening and characterization of b‐LNPs with varying N/P ratios. a) Physicochemical properties and DNA EE% of b‐LNPs at different N/P ratios. b) Schematic representation of b‐LNPs with different lipid compositions. The descriptions beneath the pie chart represent the mass ratio of ionizable lipid:phospholipid:cholesterol:PEG‐lipid. c) The particle size of synthesized b‐LNP formulations over 5 days. d) PDI value of synthesized b‐LNP formulations over 5 days. The mean ± s.d. from three independent replicates is shown.*p < 0.05, **p < 0.01, ***p < 0.001, two‐tailed *t*‐test. e) The quantification of encapsulated DNA barcodes in b‐LNP formulations with different N/P ratios over 5 days.

The b‐LNPs were synthesized by mixing 10 µm DNA barcodes in 100 mm citrate buffer (pH 4) with an organic phase containing lipids and DTX. The volume of the aqueous phase was maintained at least ten times greater than that of the organic phase to ensure the sequential precipitation of DTX and lipid components. The synthesized LNPs were then dialyzed against 1 × phosphate buffer saline (PBS) overnight at 4 °C and characterized. As shown in **Figure** **1**a, the size and polydispersity index (PDI) of all b‐LNPs synthesized exhibited favorable characteristics, with sizes ranging from 80 to 90 nm and PDI values below 0.2. Additionally, all formulations were negatively charged, with zeta potential values around −15 mV. During the screening procedure, the concentration of DNA barcodes in the aqueous phase was kept constant at 10 µm, and the drug loading was maintained at 30%. Formulations with varying N/P ratios were achieved by altering the proportion of ionizable lipids, while the input amount of DNA barcodes remained unchanged (Figure 1b).

The encapsulation efficiency (EE%) of DNA barcodes was assessed using the RiboGreen^®^ assay because a stable and high encapsulation efficiency is crucial for ensuring a detectable signal of DNA barcodes in b‐LNPs for future in vitro and in vivo experiments. As expected, an increase of the N/P ratio from 1 to 2 resulted in higher EE%, indicating that a low amount of ionizable lipid was insufficient to fully encapsulate the DNA barcodes. Maximum encapsulation efficiency peaked at ≈90% and was achieved at N/P ratios ≥3, with a slight decline at N/P = 6. In terms of stability (Figure 1c,d), all b‐LNP formulations remained stable at 4 °C over 5 days, except the formulation with an N/P ratio of 2, which exhibited increased PDI values. These stability results are consistent with prior studies on LNPs with tunable drug loadings synthesized using the concentration‐controlled sequential nanoprecipitation method.^[^ ^9^ ^]^ By quantifying the mass of DNA barcodes encapsulated within the b‐LNPs over 5 days, it was observed that the formulations with N/P ratios of 1 and 2 showed a loss in the amount of DNA barcodes encapsulated. In contrast, formulations with N/P ratios between 3 and 5 demonstrated the highest encapsulation efficiencies ≈90%, and more stable DNA barcode cargo levels at ≈4000 ng (Figure 1e). Based on these screening results, formulations with N/P ratios between 3 and 5 were identified as optimal for DNA encapsulation. To minimize the use of ionizable lipids, formulations with an N/P ratio of 3 were selected as the final optimized formulations for subsequent experiments.

### 2.2 Synthesis and Characterization of b‐LNPs with Different Drug Loadings

**Figure 2:**
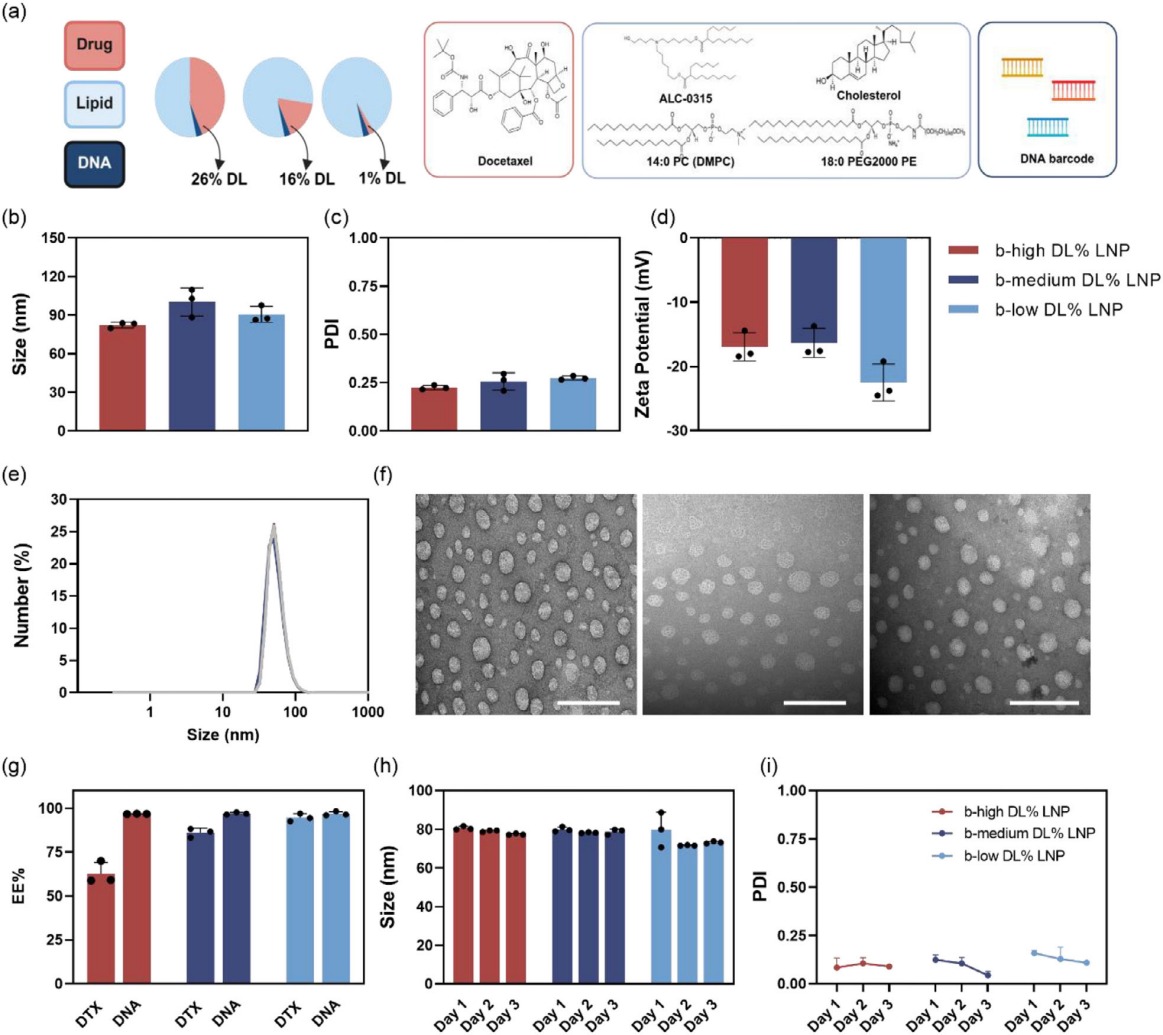
Characterization of b‐LNPs with high, medium, and low drug loadings. a) Schematic illustration of materials and compositions involved in LNP formulations. b) Size of the three b‐LNPs with different drug loadings. c) PDI values of the three b‐LNPs. d) Surface charges of the b‐LNPs. e) Size intensity distributions of the b‐LNPs. f) Their particle morphology by TEM. From left to right: b‐LNPs with low, medium, and high DL%. Scale bar = 100 nm (*n* = 3). g) The DNA barcode EE% and drug (DTX) EE% of each formulation. h) Size stability of b‐LNPs at 4 °C, over 3 days. i) PDI values of b‐LNPs at 4 °C, over 3 days. The mean ± s.d. from three independent replicates is shown.*p < 0.05, **p < 0.01, ***p < 0.001, two‐tailed *t*‐test.

To synthesize b‐LNPs with different drug loadings, b‐LNPs with high, medium, and low drug loadings were prepared using an optimized N/P ratio of 3, as described above (**Figure** **2**a). In this process, the amount of DNA barcode remained constant, while the drug‐to‐lipid ratio was adjusted to achieve the desired drug loadings. Three distinct 100 base pairs (bp) and double‐stranded (ds) DNA barcodes (Table S1, Supporting Information) were incorporated into each formulation to represent the three b‐LNPs with three different theoretical drug loadings (40%, 20%, and 1%). As demonstrated in Figure 2b–d, the sizes of the b‐LNPs were similar at ≈80 nm across different drug loadings, with similar PDI values of ≈0.2. This confirms the uniformity of the b‐LNPs. The surface charges of LNPs with different drug loadings were also similar, ranging from −20 to −15 mV. Figure 2e illustrates the size distribution of b‐LNPs with different drug loadings in 1× PBS buffer. The nearly overlapping sharp peaks for these three NPs suggest that drug loadings did not affect the size or uniformity of the b‐LNPs. The spherical morphologies of the synthesized b‐LNPs with different drug loadings were observed using a transmission electron microscope (TEM). Typically, the sizes of LNPs observed by TEM (≈50 nm) were smaller than the hydrodynamic sizes measured by DLS due to the presence of a hydration layer (Figure 2f). With an N/P of 3, the b‐LNPs with different drug loadings exhibited similar EE% of DNA barcodes ≈90%, and their sizes and PDI values remained stable over 3 days, as shown in Figure 2g–i. Interestingly, while DNA encapsulation remained consistent, the DTX EE% varied with drug input. Specifically, b‐LNPs with low DTX loading showed the highest EE% (90%), followed by b‐medium DL% LNPs (EE% = 80%) and b‐high DL% LNPs (EE% = 65%). The incorporation of positively charged ionizable lipids increases the hydrophilicity of the LNPs, which in turn limits the encapsulation capacity for hydrophobic drugs such as DTX. Despite this, the final DL% of each formulation is 26%, 16%, and 1% for high, medium, and low DL% formulations, respectively, remaining sufficient to support the central aim of this study.

Overall, the optimized b‐LNP formulation with an N/P ratio of 3 effectively produced LNPs with consistent physicochemical properties and high DNA barcode EE% across different drug loadings, demonstrating the robustness of the synthesis methods for further applications.

### 2.3 Cellular Uptake of b‐LNPs in 4T1 Cells and Bone Marrow‐Derived Macrophages (BMDMs)

Traditionally, the most common approach for tracking the delivery behaviors of NPs involves the use of fluorescent dyes, either to label the particles directly or to act as drug surrogates for detection. However, beyond the well‐documented issue of dye leakage, the use of dyes as drug surrogates can lead to aggregate‐induced quenching, potentially resulting in misleading data.^[^ ^19^, ^20^ ^]^

Directly labeling drug‐loaded NPs is also problematic, as anticancer drugs often cause cell death, complicating the uptake measurement, and washing steps can distort results.^[^ ^21^ ^]^ Our results further revealed the limited sensitivity of fluorescent dyes and the risk of altered NP properties due to dye incorporation (Figures S1 and S2, Supporting Information). Simultaneously tracking the performance of multiple formulations adds another layer of complexity.

**Figure 3:**
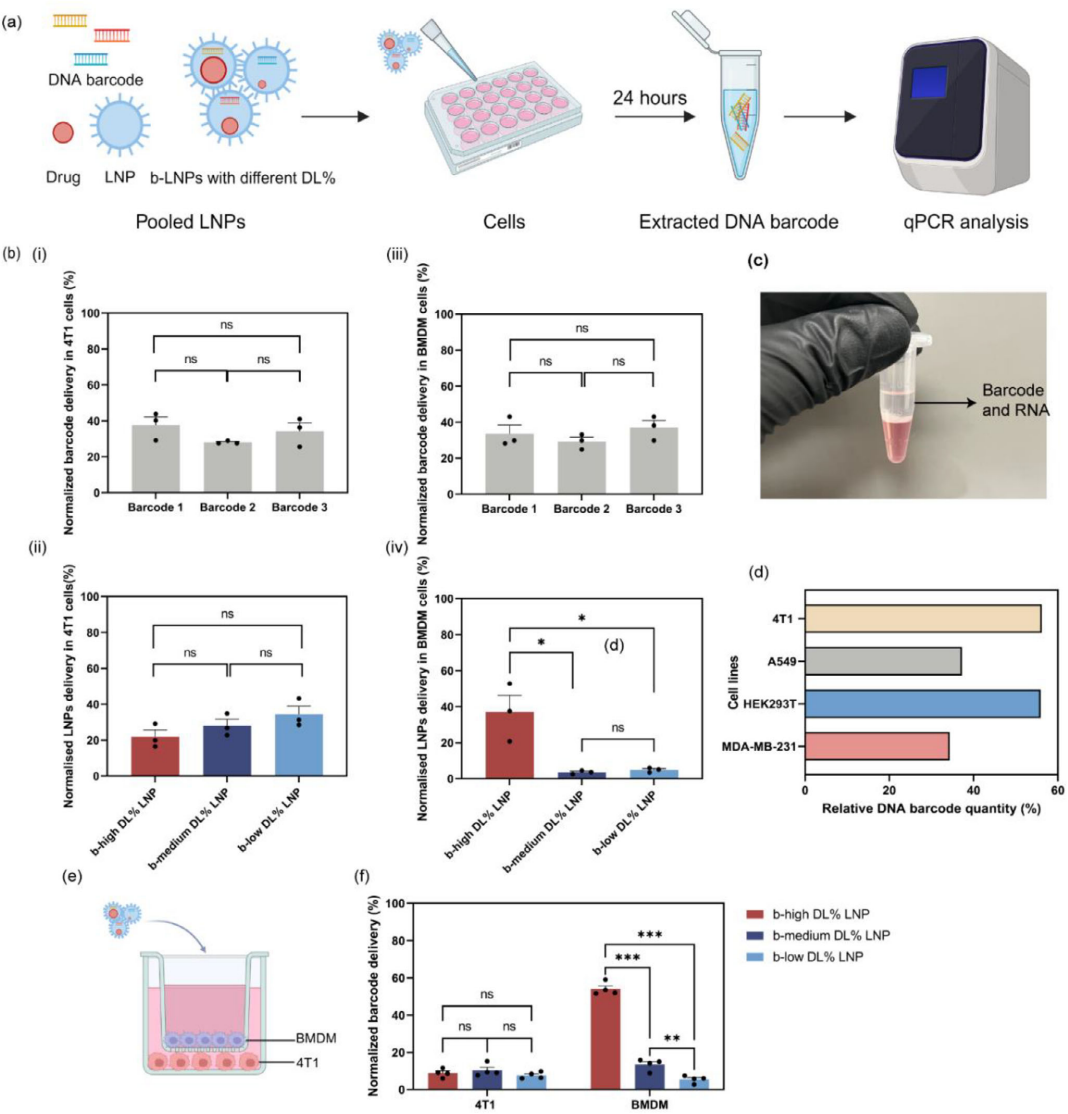
In vitro cellular uptake of b‐LNPs with different drug loadings. a) Schematic representation of the qPCR‐based workflow used to quantify cellular uptake of b‐LNPs with different drug loadings. b) Cellular uptake of mixed DNA barcodes delivered using Lipofectamine 2000: (i) in 4T1 cells and (ii) in BMDMs. Uptake efficiency of b‐LNPs with different drug loadings in (iii) 4T1 cells and (iv) BMDM cells. The prepared b‐LNPs were standardized to ensure equal levels of encapsulated DNA before experimentation. The mean ± s.d. from three independent replicates is shown.*p < 0.05, **p < 0.01, ***p < 0.001, two‐tailed *t*‐test, *n* = 3. c) Image of TRIzol‐based extraction of DNA barcodes. d) Evaluation of the TRIzol‐based DNA extraction efficiency across different cell lines. Naked DNA barcodes at a concentration of 10 µm were directly added to various cell types and immediately collected for extraction. e) Schematic illustration of cocultured BMDM and 4T1 cells in a Transwell plate with a pore size of 0.4 µm. f). Cellular uptake of b‐LNPs with different drug loadings in BMDM/4T1 cells under coculture conditions. The mean ± s.d. from three independent replicates is shown.*p < 0.05, **p < 0.01, ***p < 0.001, two‐tailed *t*‐test, *n* = 4.

To overcome these challenges, a DNA barcode was incorporated into LNPs with different drug loadings, enabling tracking of the behaviors of LNPs with different drug loadings. This approach allows for the collection of all cells, including dead cells, followed by the extraction of DNA barcodes for analysis (**Figure** **3**a). First, the in vitro performance of the designed naked DNA barcodes was evaluated to ensure that no biases existed among the three DNA barcodes. Uptake studies were conducted using 4T1 cell lines and BMDMs, representing cancer cells and tumor‐associated macrophage (TAM) in the tumor microenvironment (TME), respectively. Naked DNA barcodes were mixed with Lipofectamine 2000 and then added to 4T1 and BMDMs, with uptake quantified after 24 h of incubation (Figure 3b(i,iii)). Cells were lysed using TRIzol, and phase separation was achieved by adding chloroform. The target DNA barcodes were coprecipitated with RNA in the upper aqueous phase due to their low molecular weight (Figure 3c). After normalization to the “input” of naked DNA barcodes at 0 h, all three DNA barcodes exhibited a 40% uptake at 24 h, without significant differences in uptake efficiencies of these three DNA barcodes in 4T1 and BMDM cells. This result confirms that three DNA barcodes behaved similarly in vitro, ensuring unbiased comparisons in subsequent experiments.

The three b‐LNPs with varying drug loading but the same amount of their corresponding unique barcode were pooled together to treat 4T1 and BMDM cells. In this context, 4T1 cells served as a representative model of human TNBC cells in animal experiments, while BMDM cells acted as immune cells, serving as a model for TAMs. After 24 h of treatment with mixed b‐LNPs, the uptake results showed that there were no differences in cellular uptake of b‐LNPs with different drug loadings observed in 4T1 cells; all exhibited an uptake efficiency of ≈20–35%. (Figure 3b(ii)). This is not surprising as 4T1 cells are known as hard‐to‐transfect cell lines, and there were no significant differences in size, surface charges, morphologies, and materials used across these three formulations.^[^ ^22^ ^]^

Interestingly, BMDM preferentially internalized b‐LNPs with high drug loadings by 20‐fold compared to other formulations, despite these LNPs sharing similar properties except for drug loadings (Figure 3b(iv)). These results could be attributed to changes in other nanoparticle properties, such as stiffness and hydrophobicity, altered by drug loading. These changes may trigger different immune responses, which have previously been reported to correlate with drug loading.^[^ ^23^, ^24^, ^25^ ^]^ As a result, b‐LNPs with high drug loadings were more likely to target BMDM in this case, and there was no significant difference between the 1% and 20% drug‐loading b‐LNPs. Additionally, Figure 3d illustrates the recovery efficiency of TRIzol for different cell lines, revealing that its DNA barcode extraction capability varies depending on the cell line. When comparing the uptake efficiency of DNA barcodes across different cell lines, it is crucial to properly normalize the input to minimize the impact of varying recovery efficiencies (Figure 3d). Finally, the preferential uptake of high DL% b‐LNPs was further validated in a coculture model of BMDMs and 4T1 cells using Transwell inserts (Figure 3e). The trend persisted under coculture conditions, with high drug‐loading b‐LNPs showing significantly enhanced uptake by BMDMs (Figure 3f), further supporting the hypothesis that drug loading modulates NP–macrophage interactions.

### 2.4 Cell Enrichment Using Mixed Cell Populations

To further investigate whether LNPs with high drug loading are more favorable for macrophage uptake and to assess if different drug loadings lead to different delivery behaviors in vivo, a separation protocol was developed and validated. In this study, a cost‐effective and time‐efficient combination of Percoll gradient centrifugation and immunomagnetic beads selection method was employed as an alternative to cell sorting for isolating macrophages and cancer cells to study the tumor tropism of LNPs with different drug loadings.

**Figure 4:**
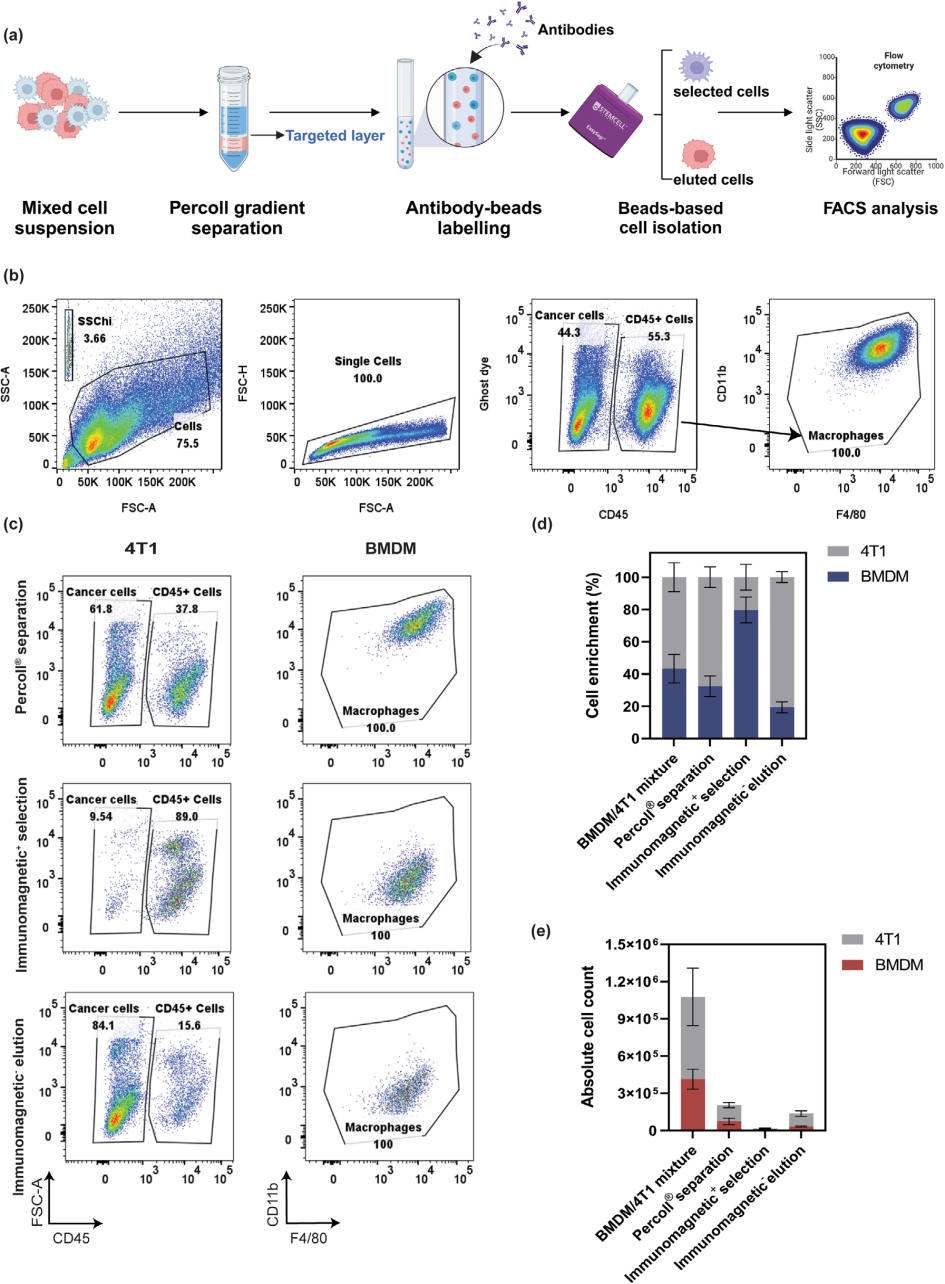
Cell enrichment of ex vivo samples using Percoll gradient centrifugation and lineage depletion through immunomagnetic selection. a) Schematic illustration of the cell enrichment process from a mixture of BMDM and 4T1 cells. b) Gating strategy used to identify and enrich target cell populations, including debris exclusion, single‐cell identification, and selection of CD45^+^, CD11b^+^, and F4/80^+^ subpopulations for BMDMs and CD45^−^ for 4T1 cells. c) Validation of cell enrichment at each stage of the process by flow cytometry after each step. d) Enrichment of cells at each step of the process. e) Absolute cell counts at each step were determined using counting beads by comparing bead‐to‐cell event ratios. The mean ± SEM from three independent replicates is shown.*p < 0.05, **p < 0.01, ***p < 0.001, two‐tailed *t*‐test.

Before conducting in vivo biodistribution experiments, the enrichment efficacy of this method was carefully examined step‐wisely on both qualification and quantification. First, this combination was tested for its ability to isolate BMDM cells from the 4T1 and BMDM cells mixture (**Figure** **4**). To be specific, Percoll gradient separation was applied to isolate the target cell layer at the upper interphase between 44% Percoll and fluorescent activated cell sorting (FACS) buffer or Dulbecco's modified Eagle medium (DMEM). Afterward, anti‐CD11b antibodies were used to label the cell suspension, with positively labeled BMDM cells retained in the column while cancer cells were collected as the eluted fraction in this case. The separation and enrichment of the cells obtained from each step were then validated using flow cytometry (Figure 4a). The gating strategy is outlined in Figure 4b, where debris was excluded through forward (FSC‐A) and side scatter (SSC‐A) parameters, so single cells were identified. The live cells were detected through the ghost dye, but at the current stage, we aimed to analyze all cells, including those killed by DTX. CD45 is a cell marker of immune cells, in this case, the BMDM cells, while CD45^−^ cells were classified as cancer cells. Further validation of BMDMs was achieved by selecting CD11b^+^ and F4/80^+^ within the CD45^+^ subpopulation. The panel used for flow cytometry is listed in Table S2 of the Supporting Information.

Figure 4c shows the validation results of cell enrichment experiments at each stage of the process. After Percoll separation, the overall quantity of cells decreased, and the percentage of the CD45^+^ population slightly decreased from 55% to 37%, potentially due to the mechanical stress from the Percoll gradient centrifugation. However, the overall results indicated successful progressive enrichment of BMDMs through Percoll separation (37.8% of BMDMs), after immunomagnetic selection (89.0% of BMDMs) and final elution yielding 84.1% 4T1 cells. The whole validation result of cell enrichment from each step is demonstrated in Figure S3 of the Supporting Information. Noticeably, the location of the BMDM population in flow cytometry tended to shift toward a more negative region, potentially caused by the changes in cell characteristics induced by bead addition. This observation requires further investigation to confirm whether the beads are correlated to cellular changes.

To determine the absolute cell amount from each step, counting beads were applied to calculate the cell numbers by comparing the ratio of bead events to cell events. The quantitative representation of cell enrichment at each stage is shown in Figure 4d, which has the same trend as the flow cytometry data. While the BMDM percentage dropped after Percoll separation, the purity of BMBM achieved 78% after antibody selection, and the 4T1 accounted for 80% of the eluted fraction, proving the overall success of the progressive enrichment. On the other hand, the cell loss from each step was also quantified in Figure 4e. The initial BMDM/4T1 mixture contained ≈1 × 10^6^ cells, with a reduction in the number of both cell types as the process progressed. The underlying reason may be attributed to the huge cell numbers of the ex vivo sample, which may exceed the capacity of the bead‐based selection kit, and the ex vivo cell compositions could contribute to the reduced efficiency of this method. Despite these limitations, the overall results still demonstrate the success of this method with high enrichment efficiency.

### 2.5 Cell Enrichment Using Mouse Tumor Tissues

**Figure 5:**
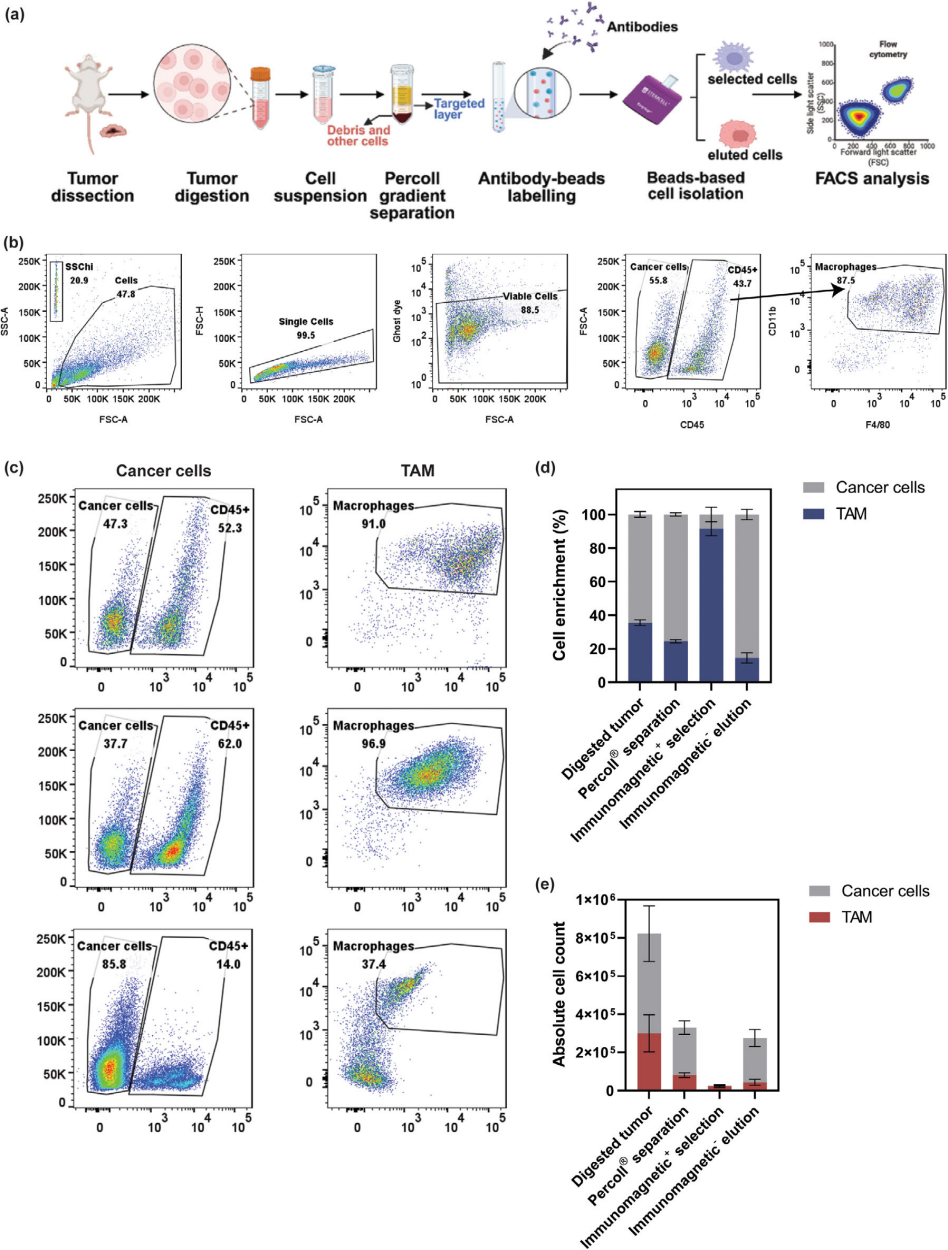
Cell enrichment of tumor samples using Percoll gradient centrifugation and immunomagnetic selection. a) Schematic illustration of the cell enrichment process from a solid tumor single‐cell suspension. b) Gating strategy for identifying and enriching target cell populations, including the separation of and selection of CD45^+^, CD11b^+^, and F4/80^+^ subpopulations for TAMs and CD45^−^ for cancer cells. c) Validation of cell enrichment by flow cytometry after each step, showing progressive separation of cancer cells and TAMs. d) Quantitative representation of cell enrichment at each step of the process. e) Absolute cell counts at each step were determined using counting bead‐to‐cell event ratios. The mean ± SEM from three independent replicates is shown.*p < 0.05, **p < 0.01, ***p < 0.001, two‐tailed *t*‐test.

To transition to in vivo experiments, we aimed to determine whether the enrichment process would be effective for in vivo experiments. Tumors from 4T1‐tumor‐bearing BALB/c female mice were dissected and digested using a blend of 1× hyaluronidase and collagenase for ≈1.5 h at 37 °C, then the resulting cell suspension was used for subsequent enrichment (**Figure** **5**a). Given the more complex composition of the TME, the validation of in vivo cell enrichment was more comprehensive than ex vivo validation. For in vivo settings, only viable cells were selected for progressive enrichment as the presence of dead cells could introduce noise into the data due to the high complexity of the tumor environment (Figure 5b).

Unlike the simple mixture of 4T1 cells and BMDM cells, the CD45^+^ subpopulation in a tumor represents not only TAMs but also other immune cells localized within TME, such as infiltrating T lymphocytes, B lymphocytes, and dendritic cells.^[^ ^26^ ^]^ As shown in Figure 5c, the percentage of TAMs within the CD45^+^ subpopulation increased from 87.5% to 91% following the Percoll separation. After immunomagnetic selection, ≈100% of the CD45^+^ cells were identified as TAMs, achieving the desired level of enrichment. Furthermore, the collected eluted fraction consisted of ≈90% cancer cells, further demonstrating the overall effectiveness of this method. Similarly, Figure 5d also illustrates a steady stepwise increase in the purity of TAMs, with the eluted fraction achieving ≈90% cancer cells. This result proves the effectiveness of this method in vivo. Interestingly, the quantitative analysis also demonstrated around half of the cell loss after Percoll separation, as shown in in vitro results, though the loss was proportionally distributed between cell types, suggesting minimal impacts on the overall outcomes (Figure 5e).

The number of TAMs accounted for 40% of the total cells in TME, which was consistent with previous reports identifying TAMs as abundantly present cell types in TNBC TME.^[^ ^27^ ^]^ The whole validation result of cell enrichment from each step is demonstrated in Figure S4 of the Supporting Information. These findings demonstrated that this combination is simple, efficient, cost‐effective, and adaptive for isolating cells both ex vivo and in vivo with less time consumed and equipment requirement. Considering the observed loss of cells from each step, this method is more suitable for highly sensitive quantification techniques such as qPCR, with fewer cells required for analysis.

### 2.6 Delivery Behaviors of b‐LNPs with Different Drug Loadings In Vivo

**Figure 6:**
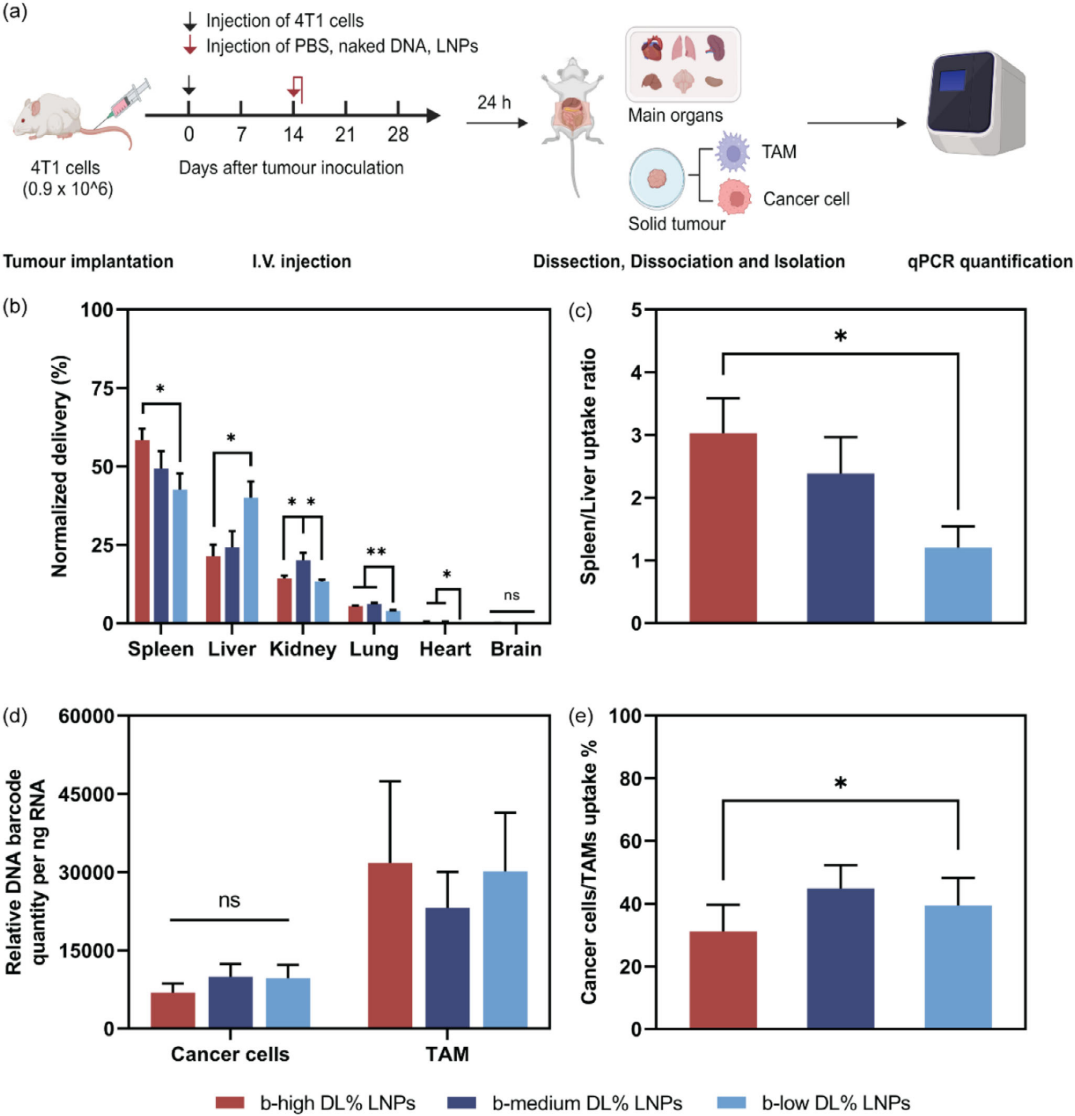
In vivo accumulation of b‐LNPs with different drug loadings in 4T1 tumor‐bearing mice. a) Schematic illustration of utilizing qPCR to probe the biodistribution and tumor tropism of b‐LNPs with different drug loadings. The b‐LNPs with high drug loading (26% DL), medium drug loading (16% DL), and low drug loading (1% DL) were pooled together and administered systemically to 4T1‐bearing BALB/c mice (*n* = 3–4). Tissues were isolated at 24 h postadministration, followed by DNA extraction, and the accumulation of b‐LNPs was quantified by qPCR. b) Biodistribution of b‐LNPs with varying drug loadings in 4T1‐bearing BALB/c mice 24 h after administration. c) Spleen‐to‐liver uptake ratio for b‐LNPs with different drug loadings. d) Accumulation of b‐LNPs with different drug loadings in cancer cells and TAMs. e) Cancer cells‐to‐TAM uptake ratio for b‐LNPs with different loadings. The mean ± SEM from three independent replicates is shown.*p < 0.05, **p < 0.01, ***p < 0.001, two‐tailed *t*‐test.

With the cell isolation and collection method established, the integration of DNA barcode into the in vivo delivery assay was employed to probe the drug loading‐delivery behavior relationship of intravenously administered LNPs. Three b‐LNPs with different drug loadings (26%, 16%, and 1%, respectively) were synthesized and pooled for injection into 4T1 tumor‐bearing BALB/c mice. Before injection, the amount of DNA barcodes encapsulated within the b‐LNPs was adjusted to the same level, with tumors of ≈250 to 300 mm^3^ on day 14 (Figure S5, Supporting Information). At 24 h postadministration, tissues of interest (liver, spleen, lung, kidney, heart, brain, and solid tumor) were isolated, and total RNA from these tissues was extracted using TRIzol. Specifically, the solid tumors were processed into single‐cell suspensions with a blend of hyaluronidase and collagenases, followed by Percoll separation and immunomagnetic bead selection to isolate TAMs and cancer cells for further analysis (**Figure** **6**a). The DNA barcodes from these extracted RNA samples were then amplified and quantified by qPCR to determine the accumulation of LNPs with different drug loadings in different tissues and to evaluate the tumor tropism of these LNPs, respectively.

Figure 6b illustrates the normalized biodistribution of b‐LNPs with different drug loadings in 4T1 tumor‐bearing mice. Overall, the b‐LNPs encapsulating DNA barcodes exhibited significantly higher accumulation across tissues compared to naked DNA barcodes (Figure S6, Supporting Information), confirming the effective encapsulation and delivery of DNA barcodes in vivo, which is consistent with our prior research.^[^ ^28^ ^]^ The biodistribution patterns of these three b‐LNPs were broadly similar due to their shared lipid composition; the majority of b‐LNPs accumulated in the spleen and liver, followed by the kidney, lung, heart, and brain. This distribution aligns with the known roles of the spleen and liver in particle filtration and clearance.^[^ ^16^, ^29^ ^]^

Notably, the distribution patterns of b‐LNPs with different drug loadings showed differences when examining tissues individually. Approximately 60% of b‐LNPs with high drug loading accumulated in the spleen, compared to ≈40% of b‐LNPs with low drug loading. Conversely, b‐LNPs with low drug loading were more liver‐targeted, with over half of these low drug loading b‐LNPs found in the liver, compared to only 20% of b‐LNPs with high and medium drug loading. This result is consistent with our expectations regarding the material burden associated with b‐LNPs with low drug loadings. b‐LNPs with medium drug loadings exhibited similar distribution as b‐LNPs with high drug loading in the liver and spleen, but exhibited higher accumulation in the kidney compared to other formulations, which is potentially related to renal clearance. All formulations displayed low accumulation in the lung and minimal levels in the heart and brain, which is expected due to the lack of filtration functions in these tissues and the protective nature of the blood–brain barrier. As illustrated in Figure 6c, b‐LNPs with high drug loading demonstrated the highest spleen‐to‐liver uptake ratio of 3, whereas b‐LNPs with low drug loading showed a spleen‐to‐liver uptake ratio of 1. This suggests that high drug‐loading b‐LNPs may preferentially interact with the spleen, potentially enhancing immune‐targeting effects for treating immune diseases and cancer,^[^ ^30^ ^]^ while b‐LNPs with low drug loading were cleared more rapidly by the liver.

As LNPs with higher DTX loading are considered stiffer, our findings are consistent with previous studies showing that stiffer NPs are preferentially taken up by the spleen, whereas softer NPs tend to readily accumulate in the liver.^[^ ^31^ ^]^ Moreover, it has been reported that an increased NP stiffness has been associated with enhanced uptake by TAMs in the TME.^[^ ^32^ ^]^ In parallel, the increased hydrophobicity of LNPs is likely to facilitate endocytosis and contribute to a more active immune response.^[^ ^33^, ^34^ ^]^ Although the precise mechanisms by which drug loading influences LNP biodistribution remain to be fully explored, current evidence strongly supports the notion that variations in drug loading alter the physicochemical and mechanical properties of LNPs, which in turn directly or indirectly impact their in vivo fate.

Next, we evaluated the accumulation profiles of these b‐LNPs at the cellular level within TME. To achieve this, DNA barcoding was extracted from TAMs and cancer cells, and total DNA barcodes in each cell type were amplified and quantified using qPCR. The relative amounts of DNA barcodes enabled direct comparisons between cell types within the TME. Figure 6d demonstrated that TAMs showed significantly higher uptake of all b‐LNP formulations compared to cancer cells. This observation is likely attributed to the high phagocytic activity and abundant presence of TAMs in the TME.^[^ ^35^ ^]^ Notably, LNPs with high drug loading exhibited the highest DNA barcode readouts in TAMs, indicating a preference for TAMs over other formulations. By targeting TAMs, therapeutic‐loading NPs achieve therapeutic efficacy indirectly through initial uptake and processing by TAMs and subsequent release of encapsulated small‐molecule drugs to kill surrounding cancer cells.^[^ ^36^ ^]^ Recent research found that the accumulation of NPs in tumors was positively correlated with the TAM density.^[^ ^36^ ^]^ All these hallmarks of TAMs provide attractive targets for cancer treatment development.

To further illustrate the preferential delivery of b‐LNPs with different drug loadings in TME, the ratio of cancer cell‐to‐TAM uptake is shown in Figure 6e, where b‐LNPs with medium and low drug loadings demonstrated a slightly higher uptake by cancer cells compared to LNPs with high drug loadings. These b‐LNPs with high drug loading showed a high TAM preference compared to other LNPs with medium and low drug loadings. Together, these results suggest that LNPs with high drug loading preferentially accumulate in TAMs compared to other formulations with lower drug loading. The delivery behaviors of LNPs in vivo were influenced by the drug loading, highlighting the potential of precisely tuning drug loading to optimize delivery strategies for enhanced treatment outcomes. For instance, NPs with high drug loading may be better suited for targeting immune cells like TAMs, whereas NPs with lower drug loading may be more effective at delivering drugs to cancer cells.

## 3 Conclusion

In this study, we established a rapid, straightforward method to investigate the in vivo delivery behaviors of LNPs with different drug loadings. By integrating DNA barcoding technology into nanoparticle formulations, a cost‐effective and robust approach has been developed to efficiently and accurately probe the drug loading–delivery behavior correlations of multiple formulations using qPCR with significantly reduced animal usage within a single experiment. Furthermore, the combination of Percoll gradient separation and immunomagnetic positive selection enabled the isolation and collection of target cell populations inexpensively and rapidly. This combination proved effective for achieving cell enrichment in both ex vivo and in vivo experiments, providing high‐quality samples for further qPCR quantification. Our approach overcomes the limitations of traditional dye‐labeling methods by offering higher sensitivity, multiplexing capabilities, and reduced reliance on cell sorters, making it an accessible and robust method for routine laboratory investigations and in vivo screening of a small library of NPs, but not limited to LNPs with different drug loadings. Our findings indicate that LNPs with high drug loading preferentially accumulate in BMDMs and TAMs compared to LNPs with lower drug loadings. Both in vitro and in vivo experiments confirmed these results, further highlighting the potential of high drug‐loading LNPs to effectively target TAMs for cancer immunotherapy in the future. Conversely, LNPs with medium and low drug loadings shared similar distribution patterns within tumors regardless of drug loading variations, suggesting that drug loading may not be the only factor influencing the uptake of TAM, but some indirect factors related to specific characteristics of high drug loading LNPs play a more important role. Moreover, the biodistribution profile further emphasized the promise of high drug‐loading LNPs for spleen‐target delivery, suggesting their utility in the development of immunomodulatory therapies. Fine‐tuning the drug loading will be a promising opportunity to optimize the target strategies within TME; for instance, high drug‐loading LNPs may be well‐suited for TAM targeting, and LNPs with lower drug loadings could be directly targeted for cancer cell targeting.

In conclusion, our method provides a practical and cost‐effective approach for studying the in vivo delivery behaviors of LNPs with different drug loadings, offering new possibilities for optimizing anticancer therapeutics delivery and in vivo formulations screening, and providing a versatile tool for broader applications such as personalized nanomedicine and TME‐targeted delivery. The investigations on LNPs with different drug loading address the critical gap in understanding the loading–delivery relationship and pave the way for future research into therapeutic efficacy and underlying mechanisms.

## 4 Experimental Section

### Materials

All chemicals were purchased and used as received unless otherwise noted. DTX was purchased from Thermo Fisher Scientific (Waltham, USA). 1,2‐dimyristoyl‐*sn*‐glycero‐3‐phosphocholine (14:0 PC DMPC) and 1,2‐distearoyl‐*sn*‐glycero‐3‐phosphoethanolamine‐*N*‐(methoxy (polyethylene glycol)‐2000) (18:0 PEG2000 PE) were obtained from Avanti Polar Lipids, Inc. (Alabaster, USA). ((4‐hydroxybutyl) azanediyl) bis (hexane‐6,1‐diyl) bis (2‐hexyldecanoate) (ALC‐0315) was purchased from SINOPEG (Xiamen, China). Quant‐iT RiboGreen assay reagents and kits were purchased from Thermo Fisher Scientific (Waltham, USA). Cholesterol and Percoll density gradient media were obtained from Sigma‐Aldrich Pty Ltd (Bayswater, Australia). Biotin anti‐mouse F4/80 antibody was acquired from Biolegend (San Diego, USA). EasySep Mouse Biotin Positive Selection Kit was purchased from STEMCELL Technologies Pty Ltd (Tullamarine, Australia). Collagenase and hyaluronidase were purchased from Sigma‐Aldrich Pty Ltd (Bayswater, Australia). 100 bp DNA barcode oligos were synthesized by Sigma‐Aldrich Pty Ltd (Bayswater, Australia). Water was obtained from a Milli‐Q system in Millipore Australia Pty Ltd (North Ryde, Australia), equipped with a 0.22 µm filter and had a resistivity greater than 18.2 MΩ cm. All other chemicals, unless specified, were purchased from Sigma‐Aldrich Pty Ltd (Bayswater, Australia).

### Methods: Synthesis and Characterization of Barcoding‐LNPs with Different Drug Loadings

b‐LNPs with different drug loadings were synthesized based on a concentration‐controlled sequential nanoprecipitation technique as previously described.^[^ ^4^ ^]^ In general, the hydrophobic drug DTX was dissolved in DMSO and other lipidic components, including ALC‐0315, DMPC, cholesterol, and DSPE‐PEG, which were dissolved in EtOH with different ratios to create an organic precursor in a glass vial. Subsequently, an aqueous phase comprising 100 mm citrate buffer (pH = 4.0) and 10 µm DNA barcode was rapidly injected into the organic precursor with no less than ten times the volume of the organic phase and then mixed under magnetic stirring with a speed of 550 revolutions per minute. This nanosuspension was subsequently dialyzed with a 10 KD molecular weight cut‐off dialysis cassette (Thermo Fisher, Waltham, USA) against 1× PBS buffer overnight at 4 °C to neutralize the pH value of the sample and to remove the residual solvents and nonencapsulated drugs. After the preparation, the resulting samples were collected for further characterization. The synthesized LNPs were stored at 4 °C for future experiments. Before in vivo administration, each b‐LNP solution was standardized to an encapsulated DNA barcode concentration of 134 ng µL^−1^ and filtered through a 0.22 µm filter to ensure sterility and remove any large aggregates. When injected intravenously, the dose of all the drugs combined was held below 5.5 mg kg^−1^ mouse body weight.

The hydrodynamic diameter, polydispersity, and zeta potential of formulated LNPs with different drug loadings were measured using a Zetasizer Nano ZS DLS system (Malvern, Worcestershire, UK) at 25 °C. LNPs were diluted 10‐fold with 1× PBS to avoid overloading the instrument and ensure accurate size measurements. Zeta potential was measured by diluting LNPs 50‐fold with Milli‐Q water to achieve the desired ion strength for measurement. The morphology and dry size of LNPs were observed using an FEI Tecnai G2 Spirit BioTwin TEM machine (Oregon, USA) operated at an accelerating voltage of 100 kV. For TEM sample preparation, 10 µL of LNPs was dropped onto a formvar‐coated copper grid. After 3 min air drying at room temperature, the redundant solution left on the grid was absorbed by a filter paper, followed by 3 min negative‐staining using 1% uranyl acetate and additional blotting with a filter paper. The EE% of DNA barcode within the LNPs was quantified using the Quant‐iT RiboGreen assay based on the standard curve of DNA barcode amount (Figure S7, Supporting Information). To determine the EE% of b‐LNPs, freshly prepared samples were ultrafiltrated using an Amicon centrifugal filter with a 100 kDa cut‐off pore size and centrifuged at 3000 × *g* for 30 min. The eluted liquid was then collected and tested for the amount of unencapsulated drug within the b‐LNPs. The drug concentration in the dissolved samples was determined using reversed‐phase high‐performance liquid chromatography (Shimadzu Corporation, Kyoto, Japan). The standard curves of different drugs in solvents are shown in Figure S8 of the Supporting Information

(1) $$\begin{array}{ccc} \mathrm{DNA}\;\mathrm{EE}\% & = & \frac{\mathrm{Total}\;\mathrm{encapsulated}\;\mathrm{DNA}\;\mathrm{amount}-\mathrm{Unen}\mathrm{caps}\mathrm{ulat}\mathrm{ed}\;\mathrm{DNA}\;\mathrm{amou}\mathrm{nt}}{\mathrm{Total}\;\mathrm{encapsulated}\;\mathrm{DNA}\;\mathrm{amount}}\\ & & \times 100\% \end{array}$$

(2) $$\mathrm{Drug}\;\mathrm{EE}\%=\frac{\mathrm{Input}\;\mathrm{drug}\;\mathrm{mass}-\mathrm{Elut}\mathrm{ed}\;\mathrm{drug}\;\mathrm{mass}}{\mathrm{Input}\;\mathrm{drug}\;\mathrm{mass}}\times 100\%$$

The stability of the b‐LNPs was monitored at room temperature for 5 days.

### Design and Synthesis of DNA Barcodes and Primers

100 bp DNA oligonucleotides with specific sequences were synthesized by Merck (The Woodlands, USA). Antisense and sense DNA oligonucleotides were resuspended to a 100 µm stock solution in nuclease‐free H_2_O or 1× Tris‐EDTA buffer and then annealed at 95 °C for 5 min to form dsDNA barcodes. These dsDNA barcodes were then transfected with Lipofectamine 3000 from Life Technologies (Carlsbad, USA) at 10 µm final concentration following the manufacturer's protocol. DNA barcodes and RNA were coextracted together using Thermofisher TRIzol reagent (Waltham, USA) following the manufacturer's instructions. The sequences of specific primers and barcodes are shown in Table S1 of the Supporting Information.

### TRIzol‐Based DNA Barcode Extraction and Quantification

The TRIzol‐based RNA and DNA barcode isolation was conducted following the manufacturer's instructions. Cells or lysed tissues were disrupted by using 0.5 to 1 mL TRIzol reagent, followed by chloroform extraction, isopropanol precipitation, and washing with 75% EtOH and drying out. The resulting RNA and DNA barcode coprecipitation pellet was resuspended with 20 to 100 µL nuclease‐free H_2_O and stored at −20 °C for further analysis. The quantification of DNA barcodes delivered in cells or tissues was performed on a Bio‐Rad CFX Connect Real‐Time PCR System (South Granville, Australia) using the PowerTrack SYBR Green Master Mix (Waltham, USA). The linear behavior of the dsDNA barcodes in qPCR was examined. The qPCR cycling conditions were 2 min at 95 °C, (15 s at 95 °C, 60 s at 60 °C) × 40 cycles. The standard curve of quantification of DNA barcodes is shown in Figure S9 of the Supporting Information.

### In Vitro Experiments Design: Cell Culture

Human lung carcinoma A549 (RRID:CVCL_0023), human kidney carcinoma HEK293T (RRID:CVCL_0063), and human TNBC MDA‐MB‐231 (RRID: CVCL_0062) cell lines were purchased from the American Type Culture Collection (ATCC, Manassas, USA). These human cell lines were cultured in DMEM supplemented with 10% fetal bovine serum (FBS), 100 U mL^−1^ penicillin, and 100 µg mL^−1^ streptomycin. The murine breast cancer 4T1 cell line (RRID:CVCL_0125) from ATCC was maintained in RPMI 1640 medium (Gibco, New York, USA) with the same supplements as human cell lines. All cells were cultured under standard conditions at 37 °C in a humidified atmosphere with 5% CO_2_ and were regularly tested to ensure they were free from mycoplasma contamination.

### Cellular Delivery of b‐LNPs with Different Drug Loadings

The barcoding formulations with high, medium, and low drug loadings were prepared and added to 6‐well plates containing BMDM cells, 4T1 cells, or cocultured BMDM and 4T1 cells at a seeding density of 1.5 to 2 × 10^6^ per well for each cell type. The cells were then incubated with the mixed b‐LNPs for 24 h at 37 °C in a 5% CO_2_ atmosphere to ensure complete LNP uptake.

To determine the initial amount of DNA barcodes introduced, 50 µL of freshly made LNPs were collected as “input” for normalization during qPCR analysis. After 24 h, the medium of each well was collected and centrifuged at a speed of 800 × *g* to harvest the floating cells. The supernatant was discarded, and the cell pellets at the bottom of the tube were retained. Then, adherent cells in the 6‐well plates were treated with 500 µL of TRIzol reagent, and the resulting cell lysates containing TRIzol were further transferred to the tubes containing cell pellets. For cells cultured on Transwell plate inserts, the cells were first detached from the insert before being treated with TRIzol. The collected samples were then stored in a −20 °C freezer for further RNA/DNA extraction procedure and qPCR analysis

(3) $$\begin{array}{ccc} \mathrm{The}\;\mathrm{recovery}\;\mathrm{efficiency}\;\left(\%\right) & = & \frac{\mathrm{The}\;\mathrm{amount}\;\mathrm{of}\;\mathrm{DNA}\;\mathrm{extracted}\;\mathrm{at}\;0\;\mathrm{hour}\;}{\mathrm{The}\;\mathrm{amount}\;\mathrm{of}\;\mathrm{initial}\;\mathrm{DNA}\;\mathrm{input}}\\ & & \times 100\% \end{array}$$

### Ex Vivo Experiments: Isolation and Culture of BMDMs

BALB/c mice were sacrificed via cervical dislocation, and their abdominal skin was carefully separated from the muscle and peeled back with a tweezer. Then the legs were dissected, and any remaining tissues were removed with a surgical blade. The joints and knuckles of the legs were cut, and the bone marrow was flushed out into a serum‐free DMEM‐containing petri dish using a 5 mL syringe with a 27‐gauge needle until the bone turned pale. Then the bone marrow‐containing medium was resuspended gently by pipetting up and down and then transferred to a new 50 mL Falcon tube, followed by centrifuging at a speed of 400 × *g* for 4 min at 4 °C. The supernatant was discarded, and 1× ACK lysing buffer (Thermo Fisher, Waltham, USA) was added to the red pellet and incubated at room temperature for 5 min. Then the reaction was stopped by resuspending the cells with iced‐cold FACS buffer (PBS, 2% FBS, 1 mm EDTA) and passed through a 70 µm cell strainer (Thermo Fisher, Waltham, USA) and centrifuged again to yield white BMDM cell pellets.

BMDMs were seeded at a concentration of 1.5 × 10^6^ cells mL^−1^ in a 6‐well plate. 50 ng mL^−1^ macrophage colony‐stimulating factor (M‐CSF, PeproTech, USA) was added to the completed medium, and the medium was changed on the second day to remove the floating cells. The medium was changed every 3 days until day 7. For the cell delivery test, the cells were treated with b‐LNPs with different drug loadings and harvested with TRIzol reagent after 24 h. In terms of the macrophage enrichment procedure, the cells were detached by a cell scraper and mixed with 4T1 cells for beads‐based on isolation and flow cytometry analysis.

### Coculture of BMDMs and 4T1 Cells

BMDMs were isolated and cultured separately for 7 days in a complete RPMI 1640 medium supplemented with M‐CSF. For coculture, BMDMs were seeded into the inserts of a 6‐well plate Transwell plate with a pore size of 0.4 µm (Corning, NY, USA) at a density of 1.5 × 10^6^ cells mL^−1^. Simultaneously, 4T1 cells were seeded on the bottom of the Transwell plate with a density of 1 × 10^6^ cells per well. The two cell types were then cocultured for 2 days.

### Animal Experiments

6‐ to 8‐week‐old female BALB/c mice were purchased from OZgene ARC (Bentley, Australia). The mice were housed in individually ventilated cages with food and water provided. All animal experiments were conducted according to the Australian Code for the Care and Use of Animals for Scientific Purposes. Experimental procedures were approved by the University of Adelaide Animal Ethics Committee (S‐2022‐070).

### Xenograft Models

4T1 tumors were inoculated in BALB/c mice by subcutaneous injection. A suspension of 0.9 × 10^6^ 4T1 cells in sterile Hank's Balanced Salt Solution was injected into the 4th mammary fat pad of BALB/c female mice under anesthesia. Mice were monitored daily, and tumor measurements were taken daily using calipers until tumors reached a size of 250 mm^3^. The size of the tumor (mm^3^) = length (mm) × width (mm^2^) × 0.5. The mice in the experimental group were administered intravenously via the tail vein with a total dose of 13.4 µg DNA barcode and 100 µg DTX‐loaded LNPs. Control mice were sham‐vaccinated with PBS or naked DNA barcodes. 24 h post‐administration, all mice were euthanized and tumors were collected in ice‐cold FACS buffer for further digestion and cell isolation, other main organs (liver, spleen, kidney, lung, heart, and brain) were collected and weighed, followed by being stored at −80 °C for subsequent DNA extraction and qPCR analysis. At least *n* = 3–4 mice per group were used for all studies, unless noted otherwise.

### Tumor Dissociation and Digestion

Tumors were placed in a small plastic dish and chopped into 2 to 4 mm long pieces, then further minced with a scalpel while being moistened with a serum‐free cell medium. Then, minced tumors were transferred to a 15 mL Falcon tube and incubated with 1× collagenase‐hyaluronidase (Sigma, Bayswater, Australia) cell culture medium solution at 37 °C for 60 to 100 min. For every 15 min, the tumors were disrupted by pipetting up and down at least ten times until complete digestion was achieved. Complete digestion was indicated by a cloudy suspension. Then, tumors were washed by adding at least 10 mL of ice‐cold FACS buffer to terminate the digestion. Then, the suspensions were filtered into 50 mL Falcon tubes using a 70 µm sterile cell strainer, followed by centrifugation at 450 × *g* for 5 min at 4 °C. The supernatant was discarded, the pellets were washed twice with 1 mm EDTA in 1× PBS, and the cell pellets were resuspended in 20 mL FACS buffer for Percoll gradient density.

### Percoll Gradient Density‐Based Cell Separation

Percoll separation has been widely adopted as a pre‐enrichment step before cell sorting or isolation to reduce the complexity of the target cell populations and remove the debris.^[^ ^27^ ^]^ To prepare gradients, 90% Percoll and 44% Percoll solutions were made according to the manufacturer's instructions by diluting with 10× PBS. For the 90% Percoll solution, 13.5 mL Percoll was diluted with 1.5 mL 10× PBS in a 50 mL Falcon tube to make a total volume of 15 mL. To make 20 mL of 44% Percoll, 9.77 mL of 90% Percoll was mixed with 10.23 mL of serum‐free DMEM in another 50 mL Falcon tube. Then, 5 mL of the 90% Percoll was withdrawn using an 18‐gauge needle and carefully and slowly injected into the bottom of the 44% Percoll until two distinct layers were observed. Next, the cell suspension was gently released on top of the 44% Percoll layer as slowly as possible. Then, this tube was centrifuged at a speed of 800 × *g* for at least 20 min at 20 °C without applying the brake. After centrifugation, two visible interphases containing cell pellets were observed. The top layer containing tumor cells and macrophages was transferred into different tubes, followed by resuspension, and washed with FACS buffer at least twice for subsequent cell enrichment and cell isolation.

### Cell Isolation Using a Magnetic‐Based Positive Selection Kit

To isolate tumor‐associated macrophages from the tumor suspension, the EasySep Mouse Biotin Positive Selection Kit was used according to the manufacturer's protocol. The cell suspension derived from Percoll gradient centrifugation was resuspended with FACS buffer to a desired cell density and incubated with biotinylated anti‐F4/80 antibodies or biotinylated anti‐CD11b antibodies supplied by BioLegend (San Diego, USA) to specifically label the macrophages. Magnetic beads were then added to the suspension, binding the biotin‐labeled cells. Then, the cell mixture was processed through an EasySep magnet, and the eluted fraction was washed and collected and resuspended with FACS buffer as tumor cells. After the magnet was removed, the immunomagnetic‐positive cells retained within the tube were also resuspended with FACS buffer for further cell enrichment.

### TAM and Cancer Cell Enrichment from Isolated Cell Suspension

The initial cell suspension, including the digested tumor suspension, post‐Percoll separation, ex vivo cell mixtures, or in vivo immunomagnetic positive selection and the eluted fractions, was resuspended in FACS buffer, and the supernatant was discarded. Then, the samples were incubated with an antibody panel (Table S2, Supporting Information) at room temperature for 30 min and followed by being washed with FACS buffer twice and resuspended with 30 µL precision count beads containing FACS buffer into 200 µL cell suspension for flow cytometry analysis

(4) $$\begin{array}{ccc} & & \mathrm{Absolute}\;\mathrm{cell}\;\mathrm{count}\left(\mathrm{cells}\;\mu L^{-1}\right)=\frac{\mathrm{Cell}\;\mathrm{count}}{\mathrm{Precision}\;\mathrm{count}\;\mathrm{beads}\;\mathrm{count}}\\ & & \times \;\mathrm{Precision}\;\mathrm{count}\;\mathrm{beads}\;\mathrm{concentration}\;\left(\mathrm{beads}\;\mu L^{-1}\right) \end{array}$$

### Data Analysis and Statistics

Statistical analysis was performed using GraphPad Prism 9 for Windows, GraphPad Software, Boston, MA, USA, www.graphpad.com. Paired two‐sided *t*‐tests, unpaired two‐sided *t*‐tests, or one‐way ANOVAs were used where appropriate. Data are plotted as mean ± standard error unless otherwise stated, with *p* < 0.001 denoted as ***, *p* < 0.01 denoted as **, *p* < 0.05 denoted as *. Values for *p* are included in the appropriate figure legend or the main text.

## Conflict of Interest

The authors declare no conflict of interest.

## Supporting information



Supporting Information

## Data Availability

The data that support the findings of this study are available from the corresponding author upon reasonable request.
