# Supplementary material for: DNA Barcoding‐Enabled Tracking of Lipid Nanoparticles: Drug‐Loading‐Dependent Biodistribution and Tumor Microenvironment Targeting
Source: Adv Healthc Mater. 2025 Jun 26;14(24):2501914. doi: 10.1002/adhm.202501914 (PMC12447019; doi:10.1002/adhm.202501914)
Supplement: Supplementary file 1 — Supporting Information [file ADHM-14-0-s001.docx]

## Supplementary information

**Table S1.** The design of DNA barcodes and primers.

| **Name** | **Sequence (5'-3')** |
| --- | --- |
| DNA barcode 01 | CTGTCATGCCTGGAGGCCTATTCGATGTAAACAGAGAAACGGTGTGAAACGGTATCGCTAAGCTGCGCCGCGGAGGAGTTGAGGGTACATAGGGCGACGA |
| DNA barcode 02 | CCACATACGCTGTCTCTGGCACGTGGATGGTTTGGAGGAATCAGATCCAAGTCTGGCCAACCTCCAAGCAGGTCTAGAGTCTAAAACAGTGGTCCCCTGC |
| DNA barcode 03 | CATGATCGTTCGCTATTCAGGGGTTGACCGACACCGGATGGCTTCTCACTTGAAGTGCTGTGCGCGACAGGGTGCGTGCACCAACCAAACCTGCTTTGAC |
| DNA barcode 01_F | CTGTCATGCCTGGAGGCCTAT |
| DNA barcode 01_R | TCGTCGCCCTATGTACCCTCA |
| DNA barcode 02_F | CACATACGCTGTCTCTGGCA |
| DNA barcode 02_R | GCAGGGGACCACTGTTTTAGA |
| DNA barcode 03_F | GATCGTTCGCTATTCAGGGG |
| DNA barcode 03_R | AAAGCAGGTTTGGTTGGTGC |


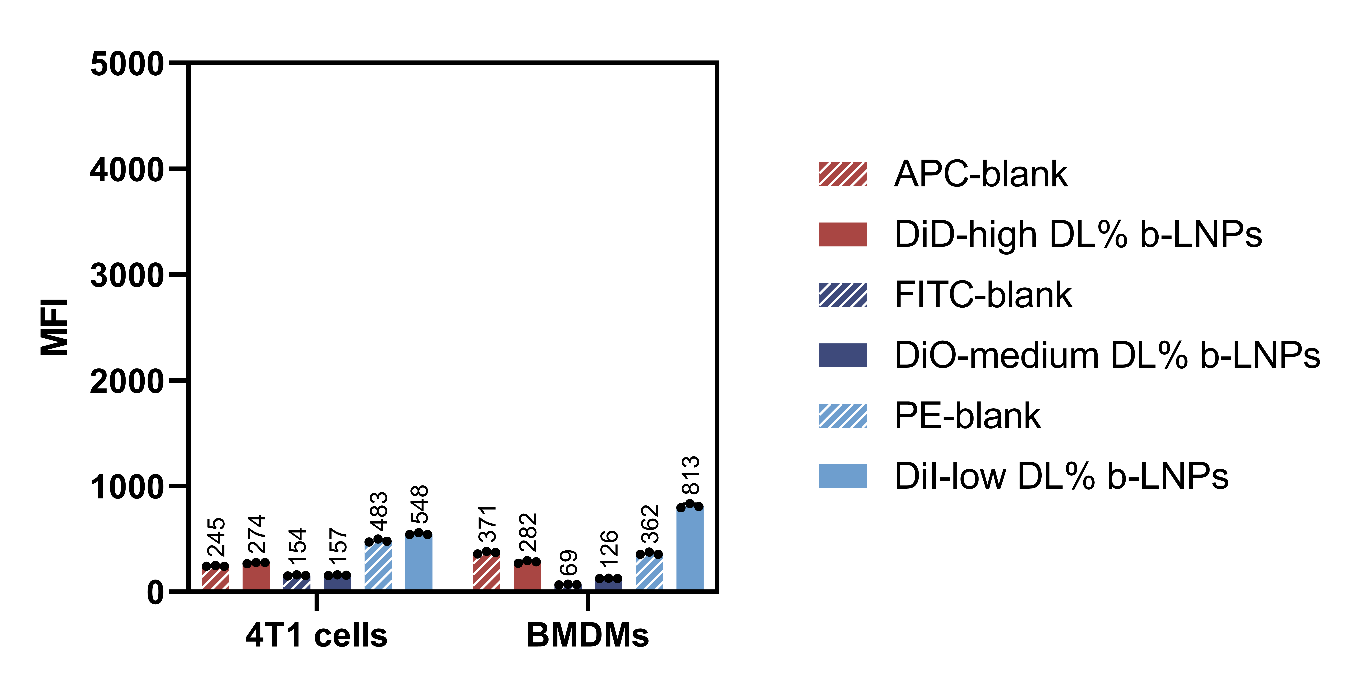


**Figure S1**. Detection limit of using fluorescence labeling for tracking b-LNPs delivery. Each fluorescent dye represents a specific b-LNP formulation with a certain drug loading: 1,1'-dioctadecyl-3,3,3',3'-tetramethylindodicarbocyanine (DiD) for high DL%, 1,1'-dioctadecyl-3,3,3',3'-tetramethylindodicarbocyanine (DiO) for medium DL%, and 1,1'-dioctadecyl-3,3,3',3'-tetramethylindocarbocyanine (DiI) for low DL%. b-LNPs were labeled with the corresponding dye at the 0.5 mol% of the total lipid content and administered to co-cultured 4T1 cells and BMDMs at the same particle dose used for DNA barcoding experiments. DiD for high drug loading LNPs, DiO for medium DL LNPs; DiI for low DL LNPs. Dye-labeled b-LNPs were mixed and incubated with co-cultured 4T1 and BMDM cells for 24 hours. Flow cytometry analyzed cellular uptake using the APC channel for DiD, FITC for DiO, and PE for DiI. Data are presented as mean ± s.d. (n = 3).


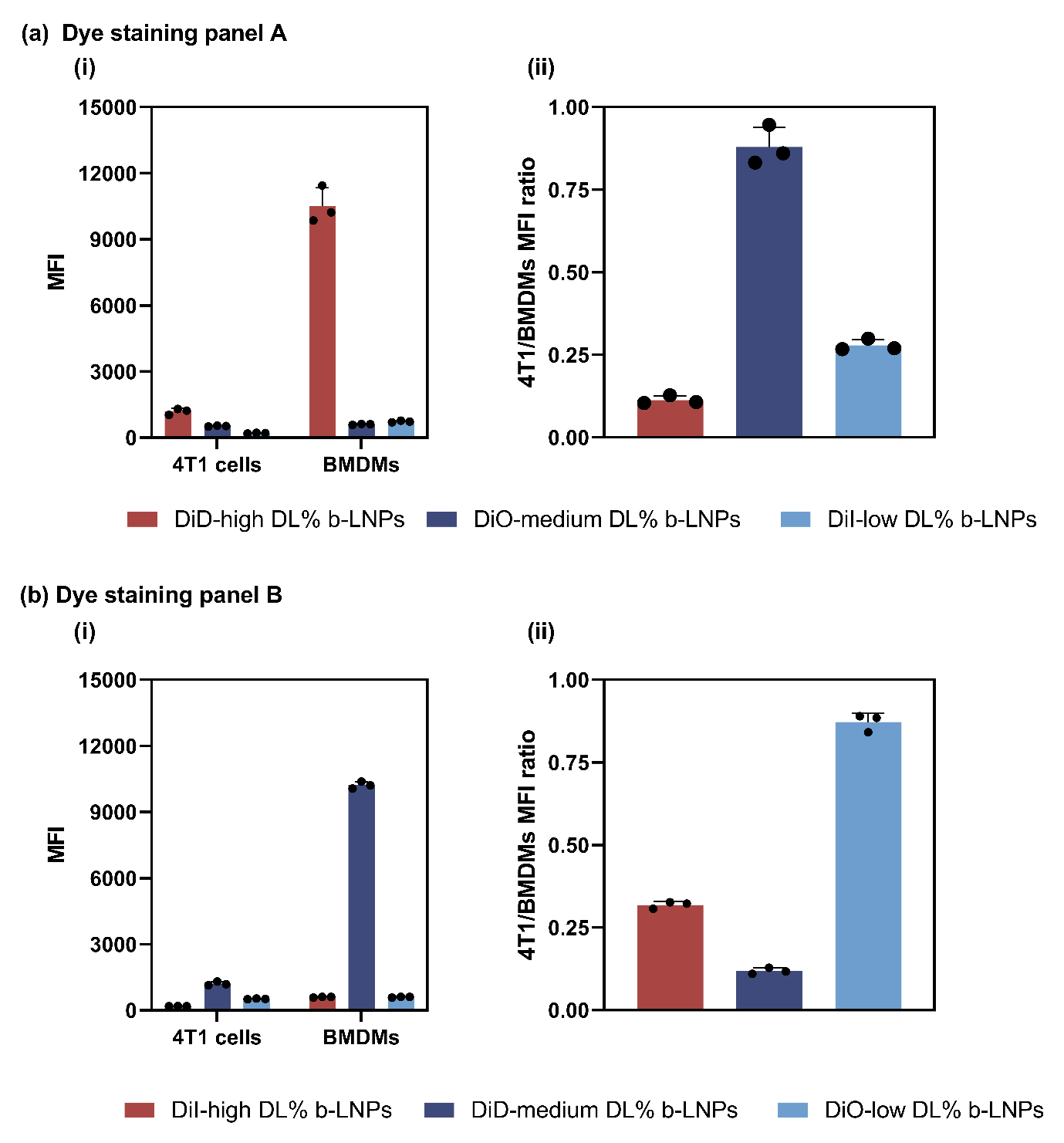

**Figure S2**. Cellular uptake of mixed b-LNP formulations with different fluorescent dyes and dye-labeling panels. (a) Uptake of b-LNPs with different drug loadings by co-cultured 4T1 cells and BMDMs, labeled using dye-labeling panel A: DiD for high drug loading (DL%) b-LNPs, DiO for medium DL% b-LNPs, and DiI for low DL% b-LNPs. (i) Median fluorescence intensity (MFI) readouts for each formulation across the two cell types; (ii) The 4T1/BMDM uptake ratio for b-LNPs with different drug loadings labeled with panel A. (b) Uptake of b-LNPs with different drug loadings by co-cultured 4T1 cells and BMDMs, labeled using dye-labeling panel B: DiI for high DL% b-LNPs, DiD for medium DL% b-LNPs, and DiO for low DL% b-LNPs. (i) MFI readouts for each formulation across the two cell types; (ii) The 4T1/BMDM uptake ratio for b-LNPs with different drug loadings labeled with panel B. The dye-labeling sequence was altered in both panels while keeping the particle dose constant. The observed differences highlight the potential influence of dye-labeling on the multiplexed uptake behaviours of b-LNP formulations. Data are presented as mean ± s.d. (n = 3).”

**Table S2**. The panel for cell enrichment using flow cytometry.

| **Antibody** | **Fluorophore** | **Dilution** | **Chanel** | **Brand** | **Catalog Number** | **Clone** | **Isotype** |
| --- | --- | --- | --- | --- | --- | --- | --- |
| L/D Ghost Dye | APC-Cy7 | 1/800 | R780 | TONBO^™^ | 13-0865-T100 |  |  |
| CD11b | Percp-Cy5.5 | 1/500 | B710 | Biolegend^®^ | 101227 | M1/70 | Rat IgG2b, k |
| F4/80 | PE-Cy7 | 1/300 | B780/YG780 | Biolegend^®^ | 123114 | BM8 | Rat IgG2a, k |
| CD45.2 | FITC | 1/200 | B530 | Biolegend^®^ | 141705 | 104 | Mouse (SJL) IgG2a, k |


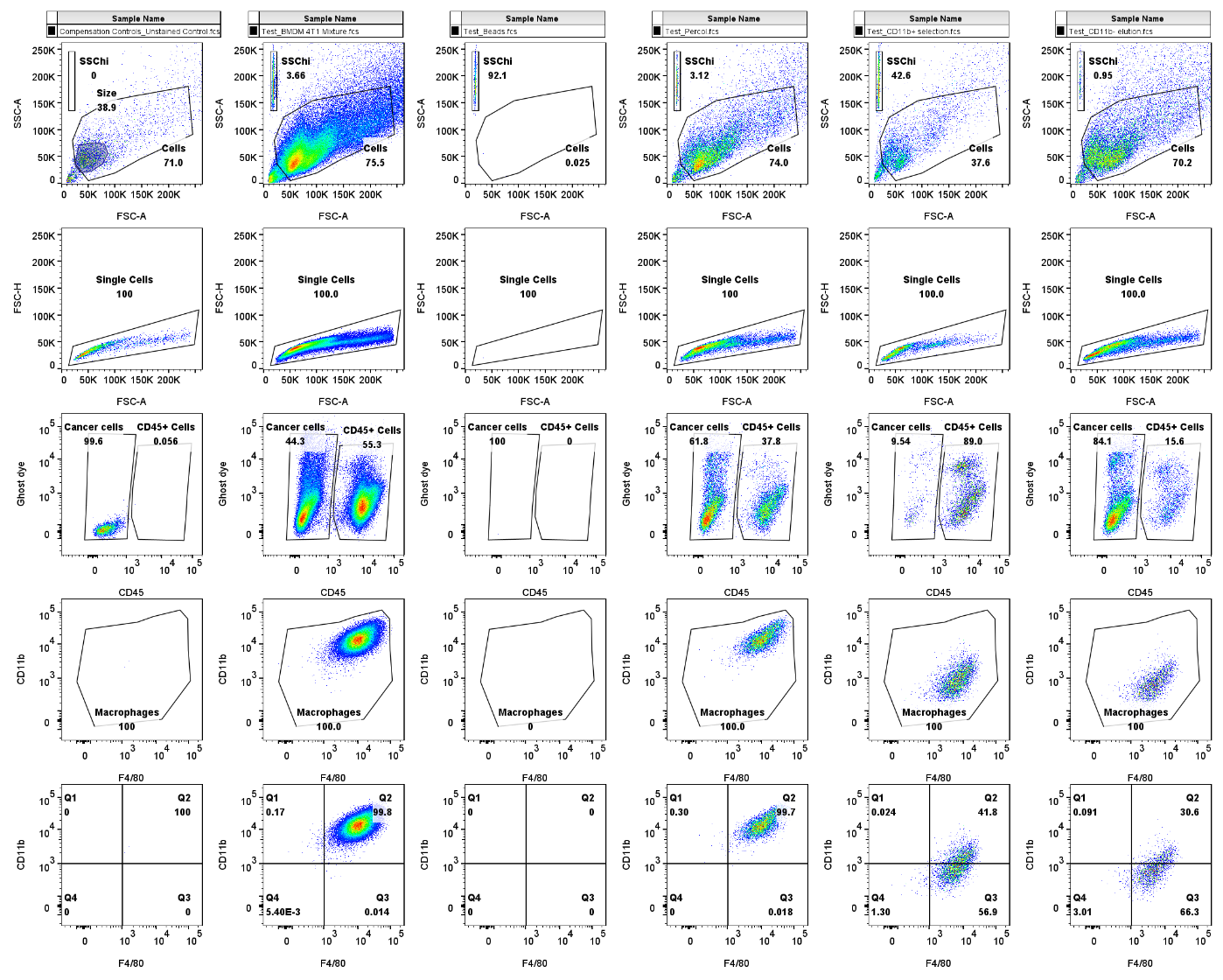


**Figure S3.** Validation of ex vivo cell enrichment using flow cytometry.


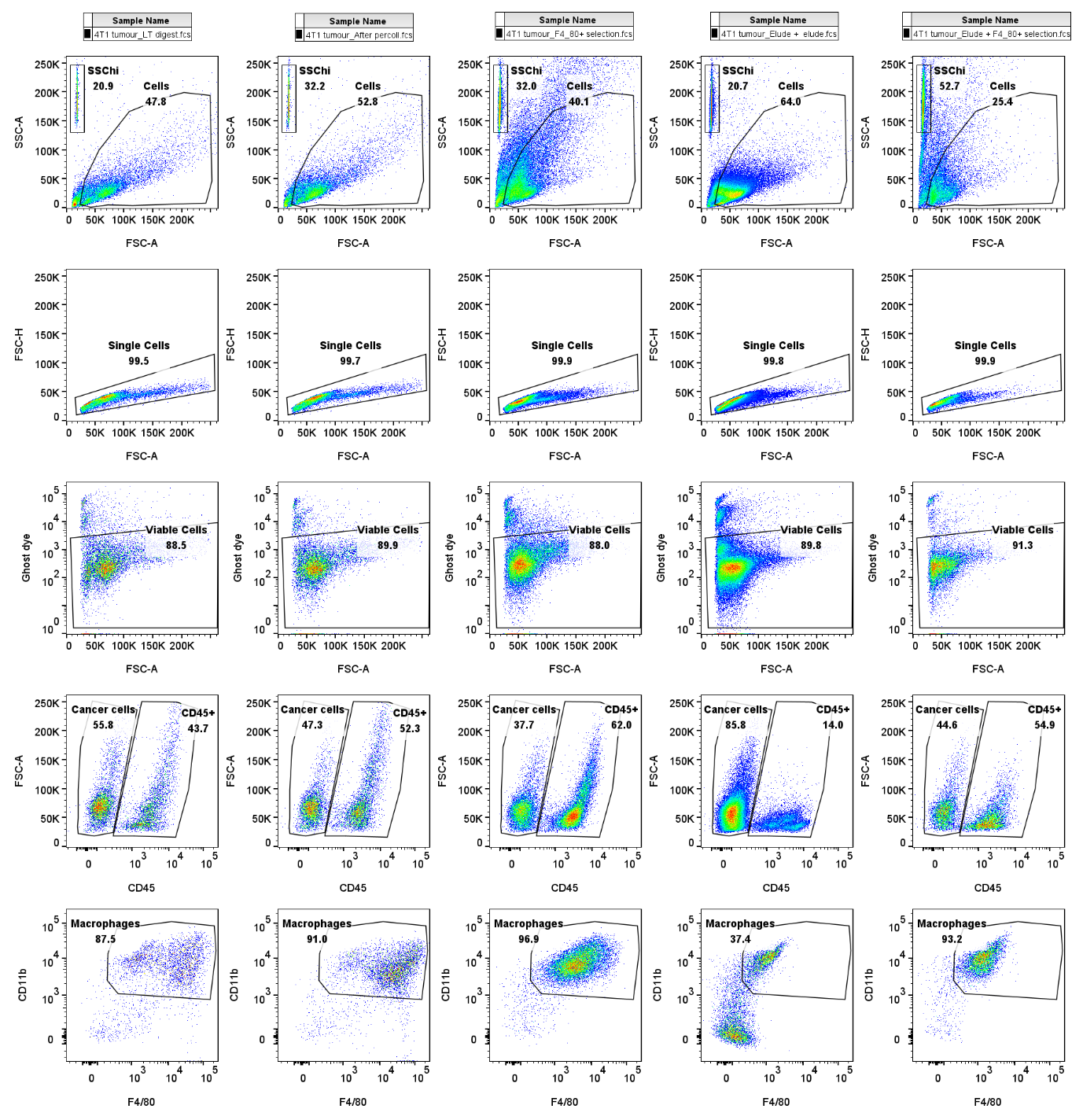


**Figure S4**. The validation of in vivo cell enrichment in flow cytometry.


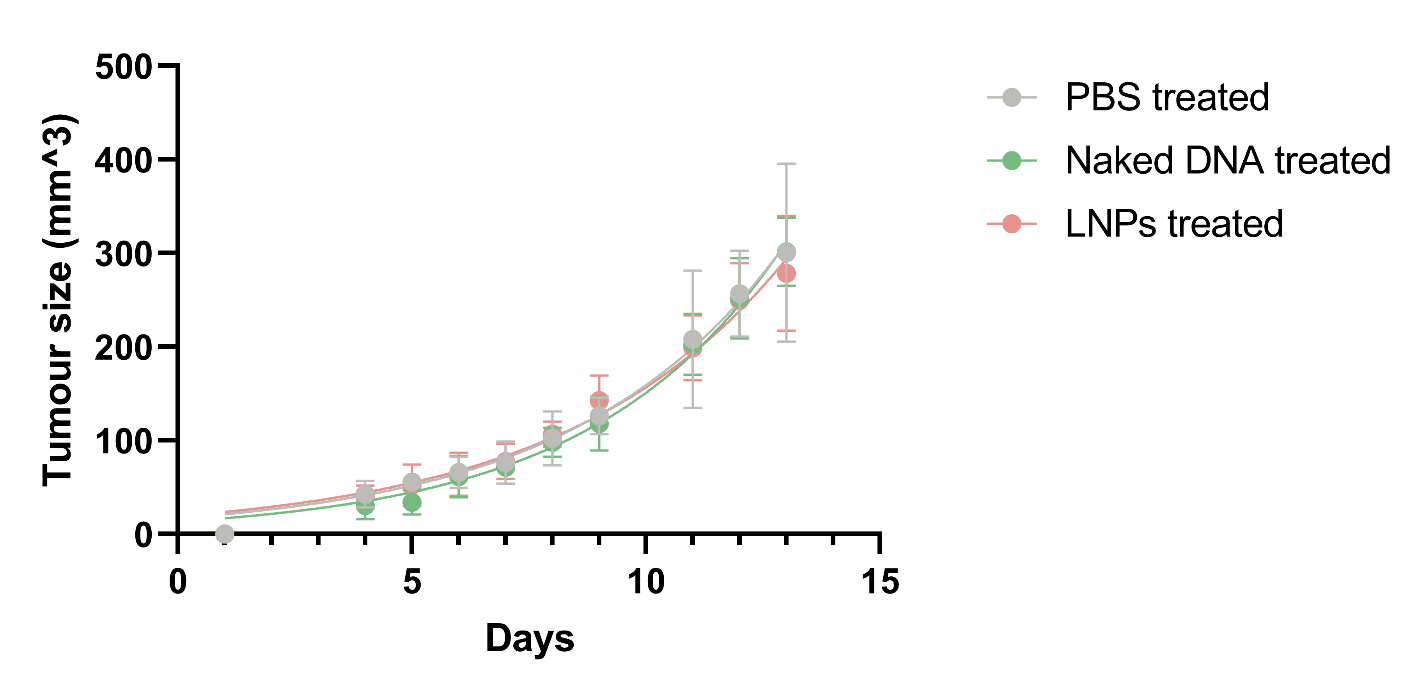


**Figure S5.** Tumor growth curve of 4T1-implanted BALB/c mice over 2 weeks.


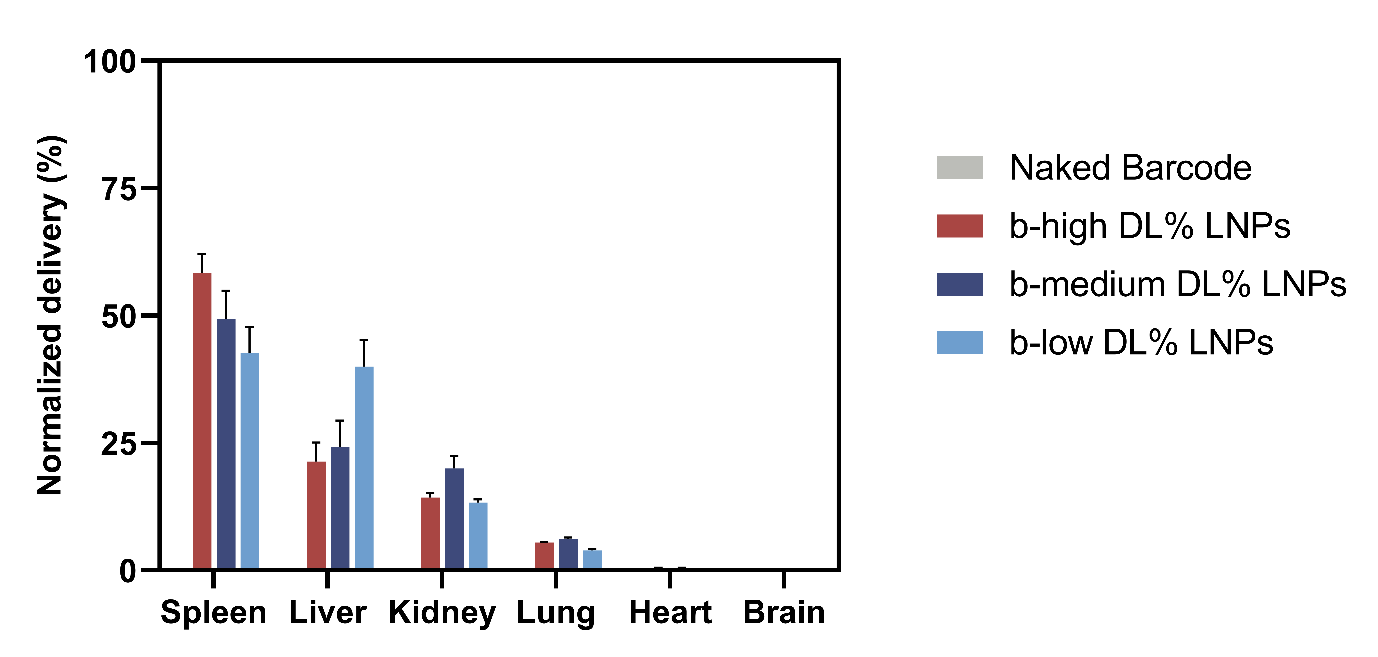


**Figure S6.** In vivo accumulation of naked DNA and b-LNPs with different drug loadings in 4T1 tumor-bearing mice.


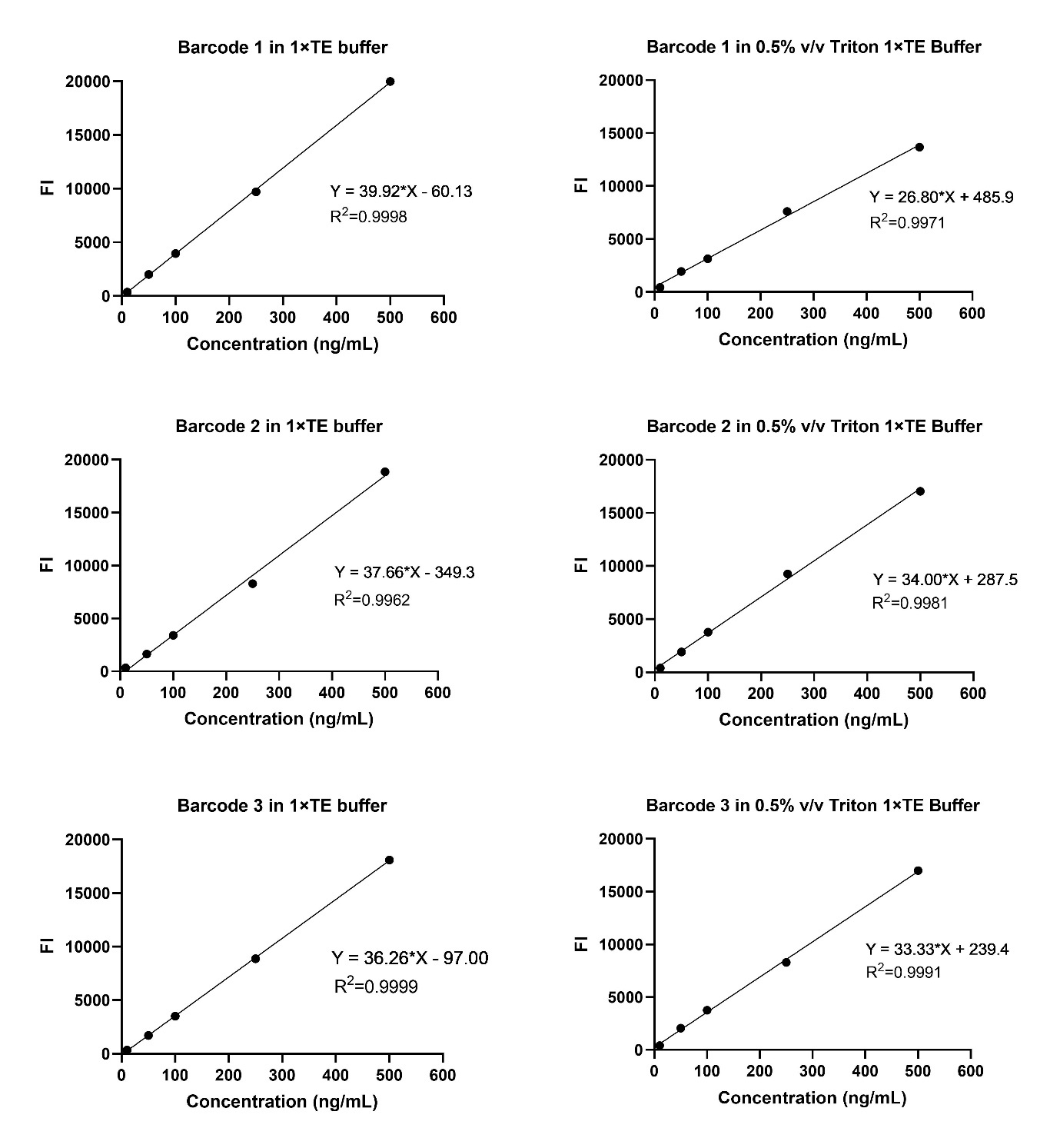


**Figure S7.** Standard curve of DNA barcode concentration versus fluorescence intensity for determining encapsulation efficiency.


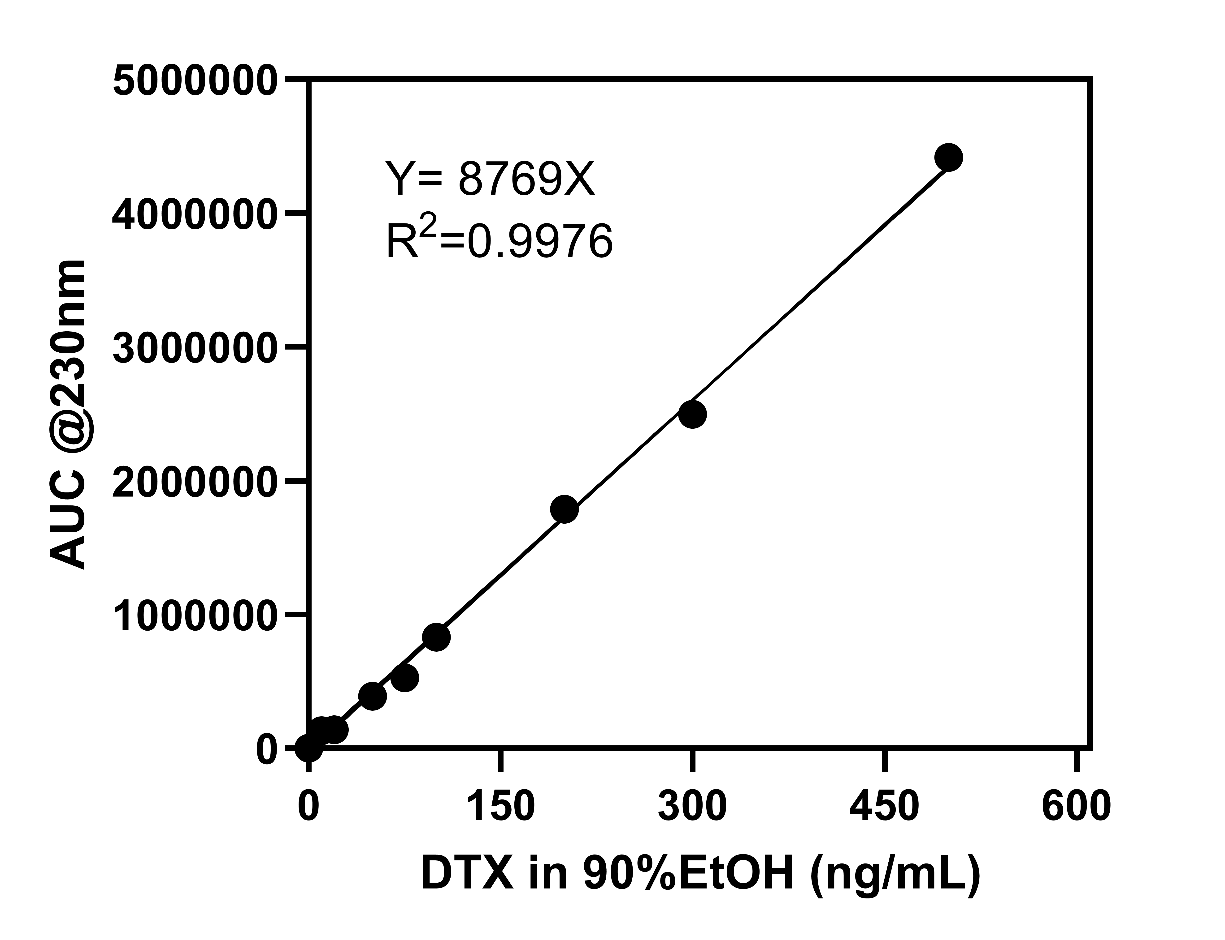


**Figure S8**. Standard curve of DTX in 90% EtOH, determined by HPLC. The mobile phase consisted of 90% acetonitrile with 0.1 % trifluoroacetic acid (TFA), and the stationary phase was Milli-Q water with 0.1% TFA. The flow rate was set at 1 mL/min.


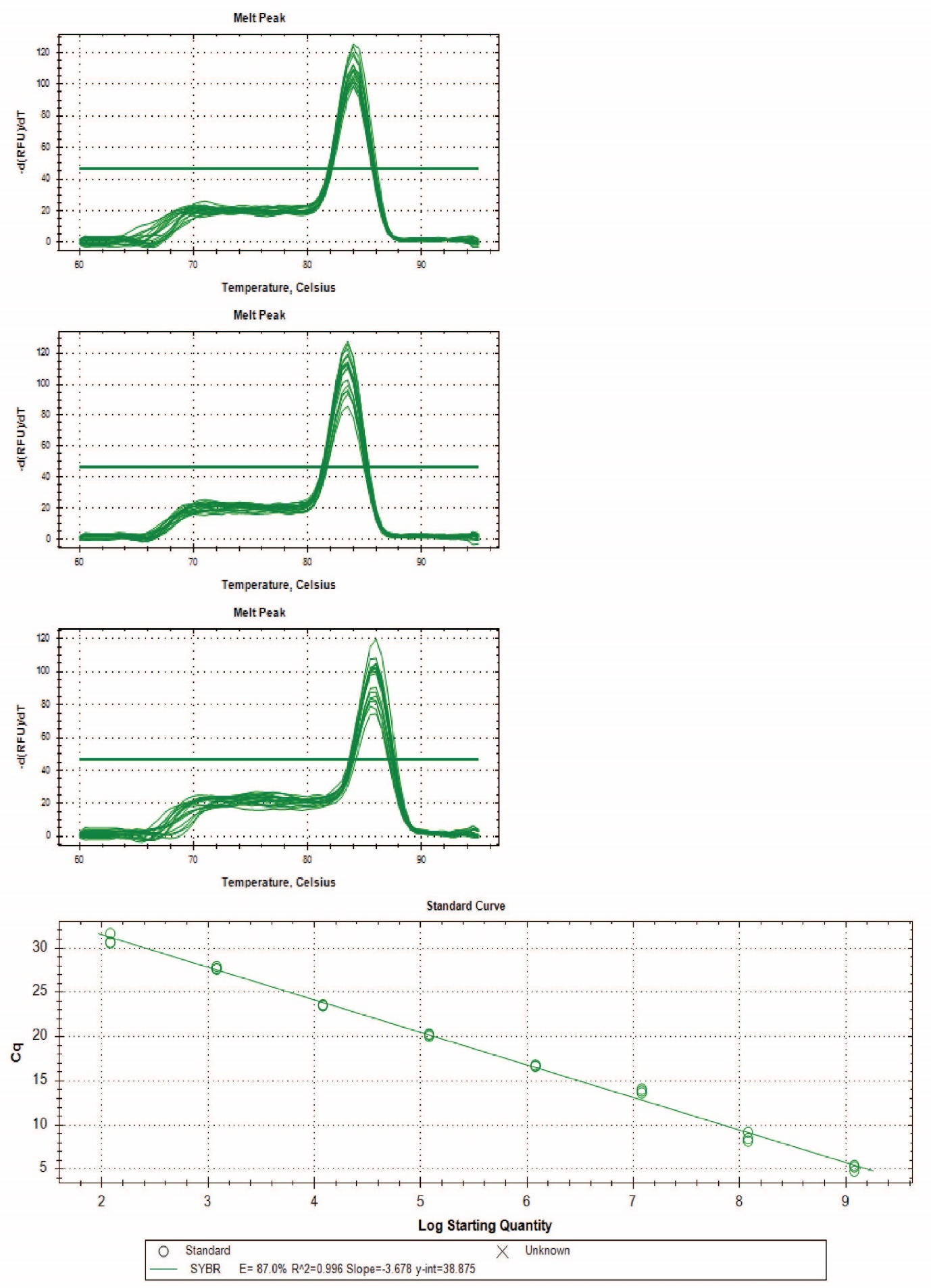


**Figure S9.** Melt peak of three DNA barcodes and standard curve of DNA barcodes for qPCR quantification.
